# The Ca^2+^–NO–ROS Crosstalk Induced by Arachidonic Acid in Human Lung Fibroblasts: Implications for Pulmonary Fibrosis

**DOI:** 10.3390/ijms27094016

**Published:** 2026-04-30

**Authors:** Karen Esmeralda Sánchez-Pluma, Edgar Martínez-Romero, José Everardo Avelino-Cruz, Giorgia Scarpellino, Valentina Brunetti, Monica Savio, Luis G. Vázquez-de-Lara Cisneros, Francesco Moccia, Roberto Berra-Romani

**Affiliations:** 1 Department of Biomedicine, School of Medicine, Benemérita Universidad Autónoma de Puebla, Puebla 72410, Mexico; karenesmeraldasanchezpluma@gmail.com (K.E.S.-P.); edgarmar.ro616@gmail.com (E.M.-R.); 2 Laboratorio de Cardiología Molecular, Instituto de Fisiología, Benemérita Universidad Autónoma de Puebla, Puebla 72560, Mexico; jose.avelino@correo.buap.mx; 3 Department of Biology and Biotechnology “L. Spallanzani”, University of Pavia, 27100 Pavia, Italy; giorgia.scarpellino@unipv.it (G.S.); valentina.brunetti01@universitadipavia.it (V.B.); 4 Immunology and General Pathology Unit, Department of Molecular Medicine, University of Pavia, 27100 Pavia, Italy; monica.savio@unipv.it; 5 Laboratorio de Medicina Experimental, Facultad de Medicina, Benemérita Universidad Autónoma de Puebla, Puebla 72420, Mexico; luis.vazquezdelara@correo.buap.mx; 6 Department of Medicine and Health Sciences “V. Tiberio”, University of Molise, 86100 Campobasso, Italy; francesco.moccia@unimol.it

**Keywords:** lung fibroblasts, arachidonic acid, calcium signalling, GPR40, reactive oxygen species, nitric oxide, store-operated calcium entry, TRPV4 channels, lysosomes

## Abstract

Arachidonic acid (AA) is an emerging regulator of fibroblast activity in pulmonary fibrosis; however, the underlying intracellular mechanisms remain unclear. This study investigated the effects of AA on the free intracellular calcium concentration ([Ca^2+^]_i_), nitric oxide (NO), and reactive oxygen species (ROS) in human WI-38 lung fibroblasts. Using fluorescent imaging and pharmacological tools, we demonstrate that AA evokes a robust, concentration-dependent increase in [Ca^2+^]_i_. This response is initiated by G protein-coupled receptor 40 (GPR40), which leads to endoplasmic reticulum Ca^2+^ release through inositol 1,4,5-trisphosphate receptors (IP_3_Rs) and lysosomal Ca^2+^ mobilisation through two-pore channels (TPCs). Sustained Ca^2+^ elevation is primarily mediated by influx through transient receptor potential vanilloid 4 (TRPV4) channels, with a minor contribution from store-operated Ca^2+^ entry. The AA-induced Ca^2+^ signal stimulates endothelial NO synthase-dependent NO production, which in turn triggers ROS generation, revealing a tightly coupled Ca^2+^–NO–ROS signalling network. Our findings identify AA as a potent modulator of Ca^2+^ and redox signalling in lung fibroblasts, and highlight GPR40, TRPV4, IP_3_Rs and lysosomal TPCs as potential therapeutic targets for intervening in pulmonary fibrosis.

## 1. Introduction

Idiopathic pulmonary fibrosis (IPF) is the most common type of interstitial lung disease, affecting around three million people worldwide. It predominantly affects older adults and is associated with a median survival rate of three to five years following diagnosis [1]. IPF is characterised by the excessive and irreversible deposition of fibrotic tissue, leading to reduced lung compliance, impaired ventilation, and hypoxaemia [2,3]. Although current antifibrotic therapies can slow disease progression, there is currently no treatment that can reverse established fibrosis [4,5]. Therefore, identifying novel molecular targets capable of preventing or limiting extracellular matrix accumulation remains a critical unmet need.

The pathogenesis of IPF is complex and not fully understood. Evidence suggests that IPF results from defective alveolar epithelium repair following repetitive or subclinical injury [1,6,7]. Genetic predisposition is also implicated in nearly 30% of cases, with variants affecting epithelial regeneration and tissue homeostasis [7,8]. These defects are exacerbated by aberrant signalling between pneumocytes, immune cells, endothelial cells, and lung fibroblasts [2,9,10]. Lung fibroblasts are central mediators of fibrosis through their capacity for extracellular matrix deposition [3].

Fibroblast activation and differentiation are regulated by autocrine and paracrine growth factors, such as vascular endothelial growth factor, fibroblast growth factor, and transforming growth factor-β [2,9,10]. Arachidonic acid (AA), a 20-carbon ω-6 polyunsaturated fatty acid that is abundant in plasma membranes, is a modulator of key cellular processes, including ion channel regulation, migration, proliferation, apoptosis, and inflammation [11,12,13]. Previous studies demonstrate that dysregulated AA metabolism contributes to the onset and progression of IPF. Consistent with this notion, an increase in the expression levels of secretory phospholipase A_2_ (PLA_2_), which cleaves AA from plasma membrane phospholipids, has been reported in IPF fibroblasts [14]. Moreover, global knockdown of cytosolic PLA_2_ attenuates lung fibrosis in mouse models of IPF [15].

Elevated AA levels have been reported in patients with IPF, likely reflecting enhanced expression of cytosolic PLA_2_ [16]. Notably, AA signalling is closely linked to cellular redox homeostasis. Beyond serving as a substrate for eicosanoid and prostaglandin synthesis, unmetabolized AA has been increasingly recognized as an upstream modulator of intracellular signalling cascades that converge on Ca^2+^-dependent pathways, which can secondarily engage oxidative and nitrosative stress mechanisms [17]. In several cell types, including pancreatic β-cells, dermal fibroblasts, adult cardiomyocytes, endothelial colony-forming cells and vascular endothelial cells, AA induces an increase in free intracellular calcium concentration ([Ca^2+^]_i_) [13,18,19,20]. In brain microvascular endothelial cells, this response arises from Ca^2+^ release from the endoplasmic reticulum (ER) via inositol 1,4,5-trisphosphate (IP_3_) receptors (IP_3_Rs), activation of lysosomal two-pore channels 1 and 2 (TPCs) and influx of extracellular Ca^2+^ through transient receptor potential vanilloid 4 (TRPV4) channels [20].

This intracellular Ca^2+^ signal can activate multiple downstream pathways, including the synthesis of nitric oxide (NO). NO is a gaseous messenger that can be produced by Ca^2+^-dependent NO synthase isoforms, including endothelial NO synthase (eNOS) and neuronal NO synthase (nNOS) [21]. Elevated nitrite and nitrate levels in bronchoalveolar lavage fluid from IPF patients indicate increased NO production [22]. Moreover, the alveolar concentration of NO has been found to correlate with the severity of the disease [22,23].

In the lung, NO regulates vascular tone, inflammation, and tissue remodelling. However, excessive NO generation may play a crucial role in fibrotic remodelling by promoting lung fibroblast proliferation and myofibroblast differentiation [22]. In addition to these effects, accumulating evidence suggests that NO can actively contribute to oxidative stress. Indeed, an increase in [Ca^2+^]_i_ can activate nicotinamide adenine dinucleotide phosphate oxidase 5 (NOX5), thereby enhancing the generation of reactive oxygen species (ROS) [24]. Consistent with this view, several studies have shown that NO itself can stimulate ROS production, suggesting a mechanistic coupling between nitrosative and oxidative signalling pathways [25,26]. Although low ROS levels can act as signalling intermediates, excessive ROS production can promote fibroblast activation [27] and the expression of pro-inflammatory cytokines via transforming growth factor-β signalling [28], contributing to IPF progression [29]. Moreover, NO can react with superoxide anion (O_2_•^−^), thereby leading to peroxynitrite (ONOO^−^) formation, which exacerbates tissue injury [30,31,32,33]. Together, these observations indicate that NO and ROS are not merely parallel by-products of a pre-existing redox imbalance but may be mechanistically integrated into a Ca^2+^-dependent signalling axis in which NO actively drives ROS generation, thereby promoting oxidative and nitrosative stress during IPF progression. Despite increasing evidence that AA and Ca^2+^ signalling contributes to fibroblast activation, the intracellular mechanisms linking unmetabolized AA to the Ca^2+^–NO–ROS signalling pathway in lung fibroblasts remain undefined.

In this study, we used fluorescent probes sensitive to Ca^2+^, NO, and ROS to investigate the molecular mechanisms underlying AA-induced Ca^2+^ signalling in foetal human lung fibroblasts (WI-38 cell line). In particular, we examined whether AA-evoked increases in [Ca^2+^]_i_ promote NO and ROS production. Our results demonstrate that unmetabolized AA elevates [Ca^2+^]_i_ via both intracellular Ca^2+^ release and extracellular Ca^2+^ influx. This elevation directly modulates NO and ROS generation, establishing AA as a pivotal regulator of intracellular Ca^2+^ dynamics and redox homeostasis. Our findings identify potential molecular targets relevant to IPF pathogenesis and reinforce the significance of AA as a modulator of profibrotic signalling pathways.

## 2. Results

### 2.1. AA Elicits a Concentration-Dependent Intracellular Ca^2+^ Response in WI-38 Human Lung Fibroblasts

**Figure 1:**
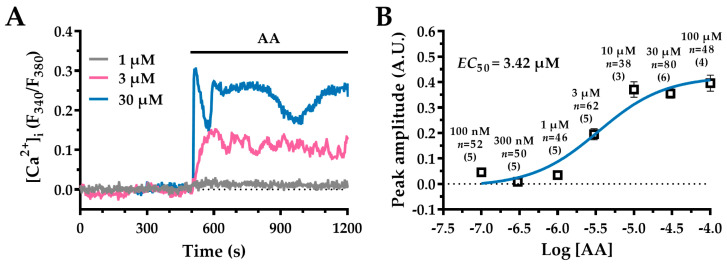
AA induces a concentration-dependent increase in [Ca^2+^]_i_ in WI-38 human lung fibroblasts. (**A**) Representative [Ca^2+^]_i_ traces from Fura-2/AM-loaded WI-38 cells exposed to increasing AA concentrations: 1 µM (grey trace), 3 µM (pink trace) and 30 µM (blue trace). In this and the following figures, the horizontal lines indicate the duration of agonist and drug application. For clarity, the baseline of each Ca^2+^ tracing has been normalised to zero. (**B**) The non-cumulative AA concentration–response relationship. The data points (squares) represent the mean ± standard error of the mean (SEM) of the [Ca^2+^]_i_ amplitudes, expressed in A.U., plotted against the logarithm of the AA concentration. The sigmoidal curve (blue line) was obtained by fitting the data to Equation (1) (see Section 4), yielding an *EC*_50_ value of 3.42 µM. The R^2^ value for the curve fit was 0.95. AA concentrations are labelled above each data point, and *n* denotes the number of cells analysed, with the number of independent experimental replicates given in parentheses.

Application of AA to Fura-2/AM-loaded WI-38 cells resulted in a concentration-dependent increase in [Ca^2+^]_i_. Figure 1A shows the time course of [Ca^2+^]_i_ in response to AA. At a concentration as low as 1 µM (grey trace), AA failed to elicit any detectable change in [Ca^2+^]_i_. However, at a concentration of 3 µM (pink trace), AA induced a slow but sustained elevation in [Ca^2+^]_i_ that persisted throughout the duration of agonist exposure. In contrast, higher concentrations (30 µM; blue trace) produced a rapid and pronounced spike in [Ca^2+^]_i_ followed by a prolonged plateau phase.

The non-cumulative AA concentration-response relationship, based on the amplitude of the [Ca^2+^]_i_ transients (Figure 1B), revealed that the threshold for a measurable effect was 3 µM (0.19 ± 0.022 A.U. (arbitrary units), *n* = 62 cells), with maximal responses achieved at concentrations exceeding 10 µM. No further enhancement of the Ca^2+^ peak was observed at 100 µM (0.39 ± 0.031 A.U., *n* = 48 cells). The half-maximal effective concentration (*EC*_50_) was calculated by fitting the concentration-response curve to Equation (1), as described in Section 4. The *EC*_50_ was found to be 3.42 µM. Notably, the coefficient of determination (R^2^) for the curve fit was 0.95. Based on these findings, 30 µM AA (0.35 ± 0.018 A.U., *n* = 80 cells) was selected for subsequent experiments due to its robust effect on Ca^2+^ signalling.

### 2.2. AA-Induced Ca^2+^ Signalling in WI-38 Human Lung Fibroblasts Is Partially Reversible and Independent of Its Metabolic Conversion

**Figure 2:**
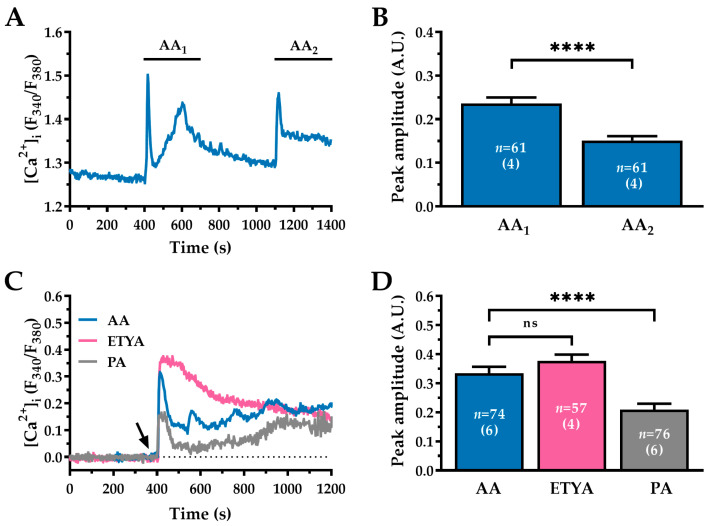
AA-induced Ca^2+^ mobilisation is specific, partially reversible and independent of AA metabolism. (**A**) Representative trace showing the [Ca^2+^]_i_ response to the sequential application of 30 µM AA with an intervening washout in physiological saline solution (PSS) (see Section 4 for composition). Note the incomplete return to baseline after the first washout. (**B**) Quantitative comparison of peak response amplitude for initial (AA_1_) and subsequent (AA_2_) AA stimulation. (**C**) Representative [Ca^2+^]_i_ traces evoked by AA (blue), its non-metabolizable analogue ETYA (pink), and the fatty acid PA (grey), each applied at 30 µM. The black arrow indicates the time of drug application, which lasts for the duration of the experiment. For clarity, the baseline of all traces has been adjusted to zero. (**D**) Mean ± SEM of peak Ca^2+^ amplitudes evoked by AA, ETYA, and PA (all at 30 µM), expressed in A.U. The *n* value indicates the number of cells analysed, with the number of independent experimental replicates used indicated in parentheses. Statistical comparisons were performed using the Wilcoxon test in panel (**B**), and the Kruskal–Wallis test in panel (**D**) (ns, *p* > 0.05; ****, *p* < 0.0001).

The Ca^2+^ response to 30 µM AA exhibited partial reversibility. Figure 2A shows a representative Ca^2+^ recording from WI-38 human lung fibroblasts exposed to two consecutive applications of 30 µM of AA. When AA was removed during the plateau phase of the first exposure (AA_1_), the [Ca^2+^]_i_ declined rapidly but failed to fully return to baseline levels, even after extensive washout. A second application of 30 µM AA (AA_2_) produced a response with similar kinetics, characterised by an initial peak followed by a plateau (Figure 2A). However, the peak amplitude was significantly lower during the second exposure (Figure 2B) (AA_1_: 0.23 ± 0.013 A.U. vs AA_2_: 0.15 ± 0.009 A.U.; *p* < 0.0001). These attenuated secondary responses could suggest either: (1) partial depletion of intracellular Ca^2+^ stores following the initial stimulation, or (2) persistent modulation of the target channel(s) or cellular Ca^2+^ clearance mechanisms induced by AA. The incomplete recovery to baseline [Ca^2+^]_i_ following washout strongly supports the latter possibility, suggesting that AA induces persistent alterations in Ca^2+^ handling, or sustained modulation of Ca^2+^-permeable channels.

Several AA metabolites, including 8,9- and 14,15-epoxyeicosatrienoic acids, prostaglandin E2, and 12-hydroperoxyeicosatetraenoic acid are known to modulate intracellular Ca^2+^ signalling [34,35,36]. Therefore, we investigated whether AA metabolism was required for the Ca^2+^ response in WI-38 cells. This was tested using the non-metabolizable AA structural analogue 5,8,11,14-eicosatetraynoic acid (ETYA; 30 µM) [37]. ETYA induced an increase in [Ca^2+^]_i_ with a peak amplitude and kinetic profile comparable to those induced by AA in matched independent experimental replicates (Figure 2C,D; AA: 0.33 ± 0.021 A.U., *n* = 74 vs. ETYA: 0.37 ± 0.021 A.U., *n* = 57; ns, *p* > 0.05). These results strongly suggest that AA triggers Ca^2+^ mobilisation in human lung fibroblasts via direct mechanisms, independently of metabolic processing.

To further characterise fatty acid specificity, we tested palmitoleic acid (PA; 30 µM), a 16-carbon, monounsaturated Ω-7 fatty acid that has previously been shown to modulate Ca^2+^ signalling in other cell types [38,39]. PA elicited significantly weaker responses than AA (AA: 0.33 ± 0.021 A.U., *n* = 74 vs. PA: 0.20 ± 0.020 A.U., *n* = 76; ****, *p* < 0.0001; see Figure 2C,D). These results demonstrate that the effects of AA on Ca^2+^ signalling in WI-38 cells are (1) mediated by the parent compound rather than metabolites, and (2) specific compared to other unsaturated fatty acids.

### 2.3. AA-Induced Ca^2+^ Signalling Involves Ca^2+^ Release from Intracellular Stores and Ca^2+^ Influx from the Extracellular Space

**Figure 3:**
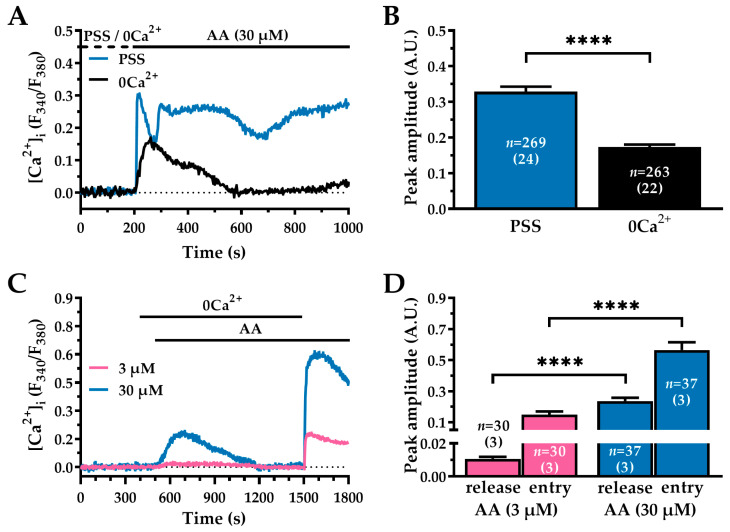
AA-induced Ca^2+^ signalling in WI-38 human lung fibroblasts involves both intracellular Ca^2+^ release and Ca^2+^ extracellular entry. (**A**) Representative Ca^2+^ traces from WI-38 cells stimulated with 30 µM AA in PSS (blue) or 0Ca^2+^ (black). In 0Ca^2+^ experiments, extracellular Ca^2+^ was removed 200 s before the application of AA to prevent Ca^2+^ entry (dotted line). For clarity, the baseline of the traces was adjusted to zero. (**B**) Mean ± SEM of peak [Ca^2+^]_i_ amplitudes obtained in 0Ca^2+^ compared to PSS, expressed in A.U. (**C**) Representative traces showing the response to different AA concentrations (3 µM, pink trace; 30 µM, blue trace) in 0Ca^2+^, followed by the restoration of extracellular Ca^2+^ in the continued presence of AA. For clarity, the baseline of the traces was adjusted to zero. The initial transient peak represents Ca^2+^ release from intracellular stores, whereas the subsequent sustained response corresponds to Ca^2+^ entry from the extracellular medium. (**D**) Mean ± SEM amplitudes of intracellular Ca^2+^ release and extracellular Ca^2+^ entry at the indicated AA concentrations, expressed in A.U. Statistical comparisons for panels (**B**,**D**) were performed using the Mann–Whitney U test (****, *p* < 0.0001). *n* denotes the number of cells analysed, and the number of independent experimental replicates used is indicated in parentheses.

The increase in [Ca^2+^]_i_ evoked by AA may arise from Ca^2+^ release from intracellular stores and/or Ca^2+^ influx across the plasma membrane [19,20,40]. To assess the contribution of each component, the AA-evoked Ca^2+^ response was measured in cells bathed either in PSS or a Ca^2+^-free solution (0Ca^2+^), which prevents Ca^2+^ entry (see Section 4 for composition; Figure 3A). Removal of extracellular Ca^2+^ (0Ca^2+^) abolished the plateau phase and significantly reduced the amplitude of the initial peak by approximately 50% (PSS: 0.32 ± 0.014 A.U., *n* = 269 vs. 0Ca^2+^: 0.17 ± 0.007 A.U., *n* = 263; ****, *p* < 0.0001; Figure 3A,B). These findings suggest that the peak response is supported by both Ca^2+^ release from intracellular stores and rapid influx of Ca^2+^, whereas the plateau phase is exclusively maintained by sustained entry of Ca^2+^ [20].

We then investigated whether the release and influx of Ca^2+^ depend on the concentration of AA by exploiting the Ca^2+^ add-back protocol. To this end, cells were first stimulated with AA in 0Ca^2+^ conditions, after which extracellular Ca^2+^ was reintroduced while AA remained present in the perfusate (Figure 3C). At a low AA concentration (3 µM; pink trace), a small transient increase in [Ca^2+^]_i_ was observed, reflecting Ca^2+^ mobilisation from intracellular stores. Upon the reintroduction of extracellular Ca^2+^, a second, sustained rise in [Ca^2+^]_i_ occurred. A similar biphasic response was observed at a higher AA concentration (30 µM; blue trace). However, the initial Ca^2+^ release from intracellular stores (AA 3 µM: 0.01 ± 0.001 A.U., *n* = 30, pink bar vs. AA 30 µM: 0.23 ± 0.021 A.U., *n* = 37, blue bar, Figure 3D) and subsequent Ca^2+^ entry (AA 3 µM: 0.14 ± 0.020 A.U., *n* = 30, pink bar vs. 0.56 ± 0.050 A.U., *n* = 37, blue bar; Figure 3D) were significantly greater at the higher AA concentration (****, *p* < 0.0001). Together, these results indicate that Ca^2+^ released from intracellular stores and Ca^2+^ influx are both dependent on AA concentration.

### 2.4. GPR40 Contributes to the AA-Evoked Ca^2+^ Response in WI-38 Human Lung Fibroblasts via Phospholipase C Activation and IP_3_Rs-Induced Ca^2+^ Release from the ER

**Figure 4:**
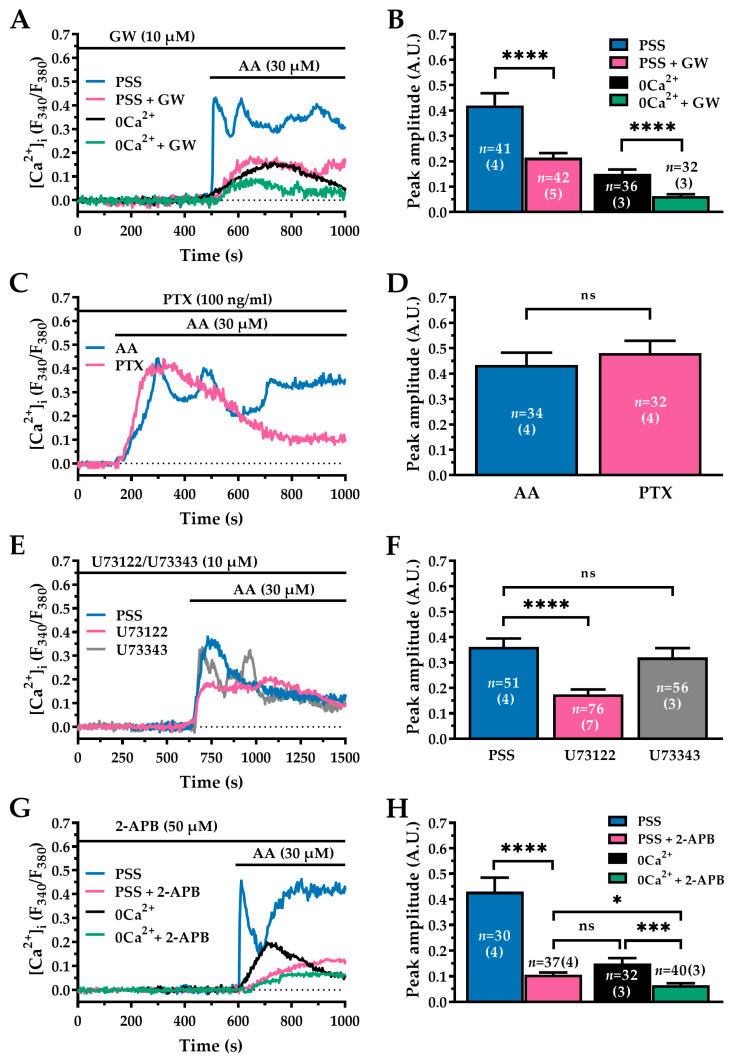
AA evokes intracellular Ca^2+^ release in WI-38 human lung fibroblasts via the GPR40–PLCβ–IP_3_Rs pathway. (**A**) Representative traces showing the Ca^2+^ response to AA (30 µM) in PSS (blue), and after pretreatment with the GPR40 antagonist GW1100 (10 µM, 10 min, PSS + GW, pink trace). The traces also show the responses to AA in a 0Ca^2+^ (black) and with GW1100 pretreatment in 0Ca^2+^ (0Ca^2+^ + GW, green trace). For clarity, the baseline of the traces was adjusted to zero. (**B**) Summary data (mean ± SEM) of peak amplitudes for the conditions shown in (**A**), expressed in A.U. Statistical analysis was performed using the Mann–Whitney U test (****, *p* < 0.0001). (**C**) Representative traces showing the response to AA (30 µM; blue) and after pretreatment with pertussis toxin (PTX; 100 ng/mL, 30 min, pink trace), a Gi/o protein inhibitor. For clarity, the baseline of the traces was adjusted to zero. (**D**) Summary data (mean ± SEM) for the conditions shown in (**C**), expressed in A.U. The Mann–Whitney U test was used (ns, *p* > 0.05). (**E**) Representative traces showing the response to AA (30 µM, blue) and following treatment with the PLC inhibitor U73122 (10 µM, 30 min, pink trace), or its inactive analogue U73343 (10 µM, 30 min, grey trace). For clarity, the baseline of the traces was adjusted to zero. (**F**) Summary data (mean ± SEM) for the conditions shown in (**E**), expressed in A.U. Statistical analysis was performed using the Kruskal–Wallis test (****, *p* < 0.0001; ns, *p* > 0.05). (**G**) Representative traces of the AA response in PSS (blue) and 0Ca^2+^ (black), and after IP_3_Rs inhibition with 2-APB (50 µM, 20 min) in PSS (PSS + 2-APB, pink trace) and 0Ca^2+^ (0Ca^2+^ + 2-APB, green trace). For clarity, the baseline of the traces was adjusted to zero. (**H**) Summary data (mean ± SEM) for conditions in (**G**), expressed in A.U. Statistical analysis was performed using the Kruskal–Wallis test (ns, *p* > 0.05; *, *p* < 0.05; ***, *p* < 0.001; ****, *p* < 0.0001). The *n* value represents the number of cells analysed, and the number of independent experimental replicates is indicated in parentheses.

G protein-coupled receptor 40 (GPR40) is a Gq protein-coupled receptor that is activated by long-chain fatty acids, including AA. It is expressed in various tissues, including the pancreas, intestine, brain, vascular endothelium, and lungs [41,42,43,44]. Herein, we first confirmed the expression of GPR40 in WI-38 cells by Western blot analysis and subsequently investigated its contribution to AA-induced Ca^2+^ signalling by pre-treating the cells with the selective GPR40 antagonist GW1100 (GW; 10 µM, 10 min) [41]. Immunoblotting confirmed that GPR40 protein is expressed in WI-38 cells (Figure S1A). In cells perfused with a normal Ca^2+^-containing solution (PSS), pretreatment with GW significantly reduced the amplitude of the Ca^2+^ peak evoked by AA (30 µM) (PSS + AA, termed PSS: 0.42 ± 0.047 A.U., *n* = 41 vs. PSS + GW: 0.21 ± 0.018 A.U., *n* = 42; ****, *p* < 0.0001; see Figure 4A,B). These results support a significant contribution of GPR40 to AA-induced Ca^2+^ signalling. However, the response was not completely abolished, suggesting the involvement of additional mechanisms. To test this hypothesis, we examined the response under 0Ca^2+^ conditions. As previously observed, removing extracellular Ca^2+^ (0Ca^2+^) markedly attenuated the AA-induced response (see Figure 3A,B and Figure 4A,B). Notably, under these 0Ca^2+^ conditions, pretreatment with GW further inhibited the remaining Ca^2+^ signal, nearly abrogating it (0Ca^2+^ + AA, termed 0Ca^2+^: 0.15 ± 0.017 A.U., *n* = 36 vs. 0Ca^2+^ + GW: 0.06 ± 0.007 A.U., *n* = 32; ****, *p* < 0.0001; Figure 4A,B). Together, these findings suggest that AA triggers at least two parallel Ca^2+^ signalling pathways in WI-38 cells: (1) GPR40–phospholipase Cβ (PLCβ)–IP_3_Rs-mediated intracellular Ca^2+^ release, and (2) activation of at least one Ca^2+^ entry pathway.

GPR40 is most associated with Gq-mediated signalling. However, there is also evidence that it can couple to Gs or Gi proteins, thereby regulating intracellular cyclic adenosine monophosphate levels [41,45]. To evaluate the potential contribution of Gi/o proteins to the AA-induced Ca^2+^ response in WI-38 cells, we treated the cells with pertussis toxin (PTX; 100 ng/mL, 30 min), a specific Gi/o protein inhibitor [46]. PTX pre-treatment did not significantly alter the amplitude of the AA-induced Ca^2+^ response (AA: 0.43 ± 0.048 A.U., *n* = 34 vs. PTX: 0.48 ± 0.048 A.U., *n* = 32; ns, *p* > 0.05; Figure 4C,D). These findings suggest that AA-induced Ca^2+^ mobilisation is independent of Gi/o signalling and is therefore most likely primarily mediated through Gq coupling.

Since Gq activation stimulates PLCβ, the selective effects of phospholipase C (PLC) inhibition were examined using U73122 (10 µM; 30 min) [47]. Subsequent application of AA (30 µM) in the presence of U73122 markedly attenuated the Ca^2+^ peak, reducing the response by approximately 60% compared to the control group (AA: 0.36 ± 0.033 A.U., *n* = 51 vs. U73122: 0.17 ± 0.019 A.U., *n* = 76; **** *p* < 0.0001; Figure 4E,F). In contrast, treatment with the inactive analogue of U73122, U73343 (10 µM; 30 min) [48], had no significant effect on the AA-induced response (AA: 0.36 ± 0.033 A.U., *n* = 51 vs. U73343: 0.32 ± 0.036 A.U., *n* = 56; ns, *p* > 0.05; Figure 4E,F), confirming that the inhibition was specific to PLC activity. Because U73122 may exert non-specific effects, including activation of Ca^2+^-permeable channels at the plasma membrane [49], additional experiments were performed under 0Ca^2+^ conditions to prevent Ca^2+^ influx. Under these conditions, the inhibitory effect of U73122 on the AA-evoked Ca^2+^ response was still observed, supporting the involvement of PLC-dependent Ca^2+^ release from intracellular stores. These results are presented in Figure S2.

Phosphatidylinositol 4,5-bisphosphate is hydrolysed by PLC to generate diacylglycerol (DAG) and IP_3_ [50]. IP_3_ binds to IP_3_Rs on the ER membrane, triggering Ca^2+^ release [51]. To confirm the involvement of this pathway, IP_3_Rs were inhibited by using 2-aminoethoxydiphenyl borate (2-APB; 50 µM, 20 min) [52]. Pre-treatment with 2-APB significantly reduced the AA-induced Ca^2+^ peak amplitude in the presence of extracellular Ca^2+^ (AA, termed PSS: 0.42 ± 0.054 A.U., *n* = 30 vs. PSS + 2-APB: 0.10 ± 0.009 A.U., *n* = 37; ****, *p* < 0.0001; Figure 4G,H) as well as in the absence of extracellular Ca^2+^ (AA + 0Ca^2+^, termed 0Ca^2+^: 0.14 ± 0.021 A.U., *n* = 32 vs. 0Ca^2+^ + 2-APB: 0.06 ± 0.008 A.U., *n* = 40; ***, *p* < 0.001; Figure 4G,H). The efficacy of 2-APB under Ca^2+^-free conditions confirms that GPR40 activation specifically mobilises Ca^2+^ from the ER via IP_3_Rs activation [53].

To evaluate whether ryanodine receptors (RyRs) contribute to AA-induced Ca^2+^ release, we used caffeine (10 mM), a well-established RyRs activator [54]. In line with our previous findings [55], application of caffeine alone failed to increase [Ca^2+^]_i_ in any of the WI-38 cells tested (50/50 cells, Figure S3A). Furthermore, pretreatment with caffeine (10 mM, 10 min) did not significantly alter the amplitude of the Ca^2+^ response evoked by AA in a Ca^2+^-free solution (0Ca^2+^: 0.18 ± 0.018 A.U., *n* = 42 vs. 0Ca^2+^ + Caff: 0.21 ± 0.014 A.U., *n* = 47; ns, *p* > 0.05; see Figure S3B,C). Consistently, the time required for the AA-induced Ca^2+^ transient to reach 63% of the maximal increase in Ca^2+^, defined as rise τ, was not significantly different from the control conditions (0Ca^2+^: 87.9 ± 13.50 s, *n* = 42 vs. 0Ca^2+^ + Caff: 114.9 ± 12.49 s, *n* = 47; ns, *p* > 0.05; see Figures S3B,D). To adopt a more cautious interpretation of these results and to directly exclude a potential contribution of RYRs, we performed additional experiments using ryanodine (Ry) at a high concentration (10 µM). Cells were incubated with Ry for 1 h prior to the experiment to lock RyRs in their non-conducting state. Under these conditions, we did not observe any significant difference in the maximal peak amplitude of the AA-evoked Ca^2+^ response (0Ca^2+^: 0.12 ± 0.011, *n* = 61 vs. 0Ca^2+^ + Ry: 0.11 ± 0.009, *n* = 55; ns, *p* > 0.05; see Figure S3E,F). These findings indicate that RyRs do not significantly contribute to AA-induced Ca^2+^ release in WI-38 cells under the experimental conditions used. Taken together, these results support the conclusion that AA-induced Ca^2+^ mobilization occurs independently of RyRs activation and is more consistent with activation of the GPR40/Gq/PLCβ/IP_3_ signalling pathway, resulting in ER Ca^2+^ release through IP_3_Rs.

### 2.5. The ER and Lysosomes Are Key Sources of AA-Induced Ca^2+^ Release in WI-38 Human Lung Fibroblasts

**Figure 5:**
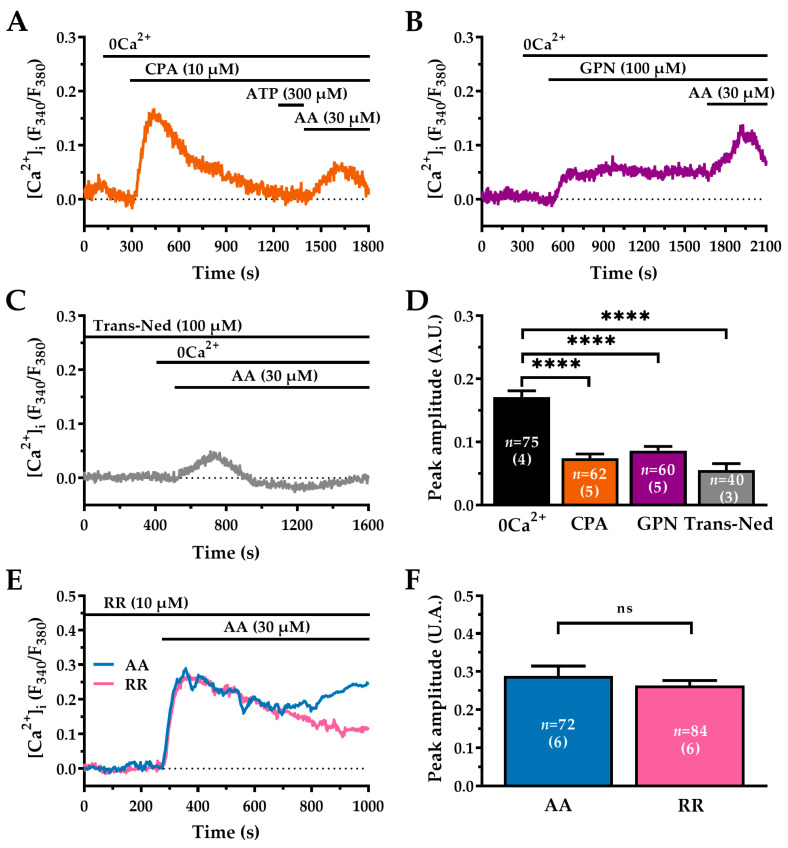
AA-induced intracellular Ca^2+^ release in WI-38 human lung fibroblasts depends on IP_3_Rs in the ER and TPCs in lysosomes, but not on mitochondrial Ca^2+^ uptake via the mitochondrial Ca^2+^ uniporter. (**A**) A representative trace showing the AA-induced Ca^2+^ signal following depletion of the ER stores with CPA (10 µM, orange trace) under 0Ca^2+^ conditions. Complete ER depletion was verified by the absence of any detectable Ca^2+^ response to 300 µM ATP. (**B**) Representative traces under 0Ca^2+^ conditions following treatment with GPN (100 µM; 20 min, purple trace), a lysosomal disruptor. (**C**) Representative traces under 0Ca^2+^ conditions following pre-incubation with the TPCs inhibitor Trans-Ned 19 (Trans-Ned; 100 µM, 45 min, grey trace). (**D**) A summary graph showing the mean ± SEM of the peak Ca^2+^ amplitudes in the cells under 0Ca^2+^ conditions: untreated control cells (black); CPA-treated cells (orange); GPN-treated cells (purple); and Trans-Ned–treated cells (grey), expressed in A.U. Statistical analysis using Kruskal–Wallis test revealed a significant reduction in Ca^2+^ release for all treatment groups compared to the control group (****, *p* < 0.0001). (**E**) Representative traces comparing control cells (blue) and cells treated with ruthenium red (RR; 10 µM, pink), an inhibitor of mitochondrial Ca^2+^ uptake. For clarity, the baseline of the traces was adjusted to zero. (**F**) Mean ± SEM peak Ca^2+^ amplitudes in control and RR-treated cells, expressed in A.U. Statistical comparison using the Mann–Whitney U test indicated no significant difference (ns, *p* > 0.05). The *n* value represents the number of cells analysed, and the number of independent experimental replicates is indicated in parentheses.

The ER serves as the main intracellular Ca^2+^ reservoir, while secondary organelles such as lysosomes and mitochondria act as auxiliary compartments that fine-tune Ca^2+^ signalling dynamics [56,57,58]. The inhibitory effects of U73122 and 2-APB indicate that the ER is targeted by AA to mobilise intraluminal Ca^2+^ through IP_3_Rs. To confirm ER involvement in the AA-induced Ca^2+^ response, we first applied cyclopiazonic acid (CPA; 10 µM), a selective inhibitor of the sarco/endoplasmic reticulum Ca^2+^-ATPase [53], under 0Ca^2+^ conditions to deplete ER Ca^2+^ stores (Figure 5A). Following 900 s of CPA pre-treatment, complete ER depletion was verified by the absence of any detectable Ca^2+^ response to adenosine triphosphate (ATP; 300 µM) in all cells tested (0/75) [59]. In WI-38 cells, ATP application in a 0Ca^2+^ medium evoked a rapid increase in [Ca^2+^]_i_ in ~74.5% of the cells tested (38/51 cells; Figure S4). In the same cells, following store depletion with 10 µM CPA, none of the cells exhibited a Ca^2+^ response to ATP (0/51), confirming that the intracellular Ca^2+^ stores had been effectively depleted (Figure S4). Upon ATP removal, subsequent exposure to AA (30 µM) in the continued presence of CPA elicited a slow, sustained increase in [Ca^2+^]_i_ (Figure 5A). However, the amplitude of this response was reduced by approximately 60% compared to control conditions (0Ca^2+^: 0.17 ± 0.009 A.U., *n* = 75 vs. 0Ca^2+^ + CPA: 0.07 ± 0.006 A.U., *n* = 62; ****, *p* < 0.0001; Figure 5A,D). While suggesting that AA can mobilise Ca^2+^ from non-ER stores, this finding demonstrates that the ER is a major contributor to AA-elicited Ca^2+^ signals.

Next, we sought to determine the contribution of lysosomes to the Ca^2+^ response evoked by AA. These organelles are a significant intracellular Ca^2+^ store, with estimated concentrations of approximately 500 µM [60], and can sustain agonist-induced Ca^2+^ release via TPCs activation [20,56,61]. To evaluate whether lysosomal integrity is required for the response, we selectively disrupted these organelles by applying Glycyl-L-phenylalanine 2-naphthylamide (GPN; 100 µM, 20 min) in 0Ca^2+^ [62,63]. This treatment elicited a transient rise in Ca^2+^, confirming successful lysosomal permeabilization and the consequent release of stored Ca^2+^ (Figure 5B). The subsequent stimulation with AA (30 µM) still triggered a Ca^2+^ response, albeit with significantly reduced amplitude compared to the control condition (0Ca^2+^: 0.17 ± 0.009 A.U., *n* = 75 vs. 0Ca^2+^ + GPN: 0.08 ± 0.006 A.U., *n* = 60; ****, *p* < 0.0001; Figure 5B,D). This suggests that, while AA can mobilise Ca^2+^ from other sources such as the ER, the lysosomal Ca^2+^ pool is essential for generating a full response. To specifically assess the involvement of TPCs, we pre-treated cells with Trans-Ned 19 (Trans-Ned; 100 µM, 45 min), a selective TPC inhibitor [53,56,64]. Application of AA (30 µM) under 0Ca^2+^ conditions only induced a modest Ca^2+^ transient (Figure 5C), whose amplitude was reduced by approximately 68% compared to the control (0Ca^2+^: 0.17 ± 0.009 A.U., *n* = 75 vs. 0Ca^2+^ + Trans-Ned: 0.05 ± 0.010 A.U., *n* = 40; ****, *p* < 0.0001; Figure 5C,D). This strong inhibition suggests that TPC-mediated lysosomal Ca^2+^ release contributes to AA-induced Ca^2+^ signalling in WI-38 fibroblasts.

Finally, we found that ruthenium red (RR; 10 µM), which is an inhibitor of the mitochondrial Ca^2+^ uniporter [65], had no effect on the Ca^2+^ response to AA (30 µM) (AA: 0.28 ± 0.026 A.U., *n* = 72 vs. AA + RR: 0.26 ± 0.013 A.U., *n* = 84; ns, *p* > 0.05; Figure 5E,F). Therefore, mitochondrial Ca^2+^ uptake does not contribute to this signal. These data indicate that intracellular Ca^2+^ release in response to AA involves the coordinated activity of ER-embedded IP_3_Rs and lysosomal TPCs.

### 2.6. AA-Induced Ca^2+^ Entry Is Largely Mediated by TRPV4 Channels, with a Smaller Contribution from Store-Operated Ca^2+^ Channels in WI-38 Human Lung Fibroblasts

Extracellular Ca^2+^ entry sustains the Ca^2+^ response to AA over time. Yang and Huang demonstrated that mouse embryonic fibroblasts express functional voltage-operated Ca^2+^ channels (VOCCs) [66]. To test the hypothesis that VOCCs mediate the sustained Ca^2+^ signal induced by AA in WI-38 cells, we employed pharmacological and functional approaches. Application of the non-specific VOCC blocker Ni^2+^ (10 µM) did not significantly alter the Ca^2+^ response to AA (AA: 0.44 ± 0.047 A.U., *n* = 30 vs. AA + Ni^2+^: 0.36 ± 0.037 A.U., *n* = 37; ns, *p* > 0.05; Figure S5A,B). However, we could not evaluate the effect of the L-type channel antagonist nifedipine (1 µM) as this elicited an unexpected Ca^2+^ signal in all cells tested (32/32) (Figure S5C). Due to the limited specificity of Ni^2+^, we investigated functional VOCC expression by depolarising the membrane with high extracellular K^+^ (20 mM). This manoeuvre failed to produce any detectable increase in cytosolic Ca^2+^ in any of the cells tested (*n* = 37; Figure S5D). This demonstrates that VOCCs are not functionally expressed in this cell line and therefore do not contribute to the AA-evoked signalling.

**Figure 6:**
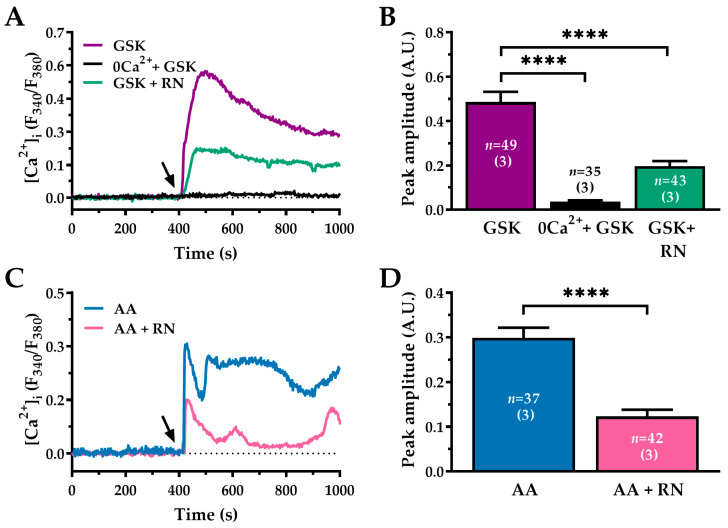
Functional expression of TRPV4 channels and their contribution to AA-induced Ca^2+^ signalling in WI-38 human lung fibroblasts. (**A**) Representative traces showing increases in [Ca^2+^]_i_, evoked by the selective TRPV4 agonist GSK1016790A in PSS (GSK; 20 nM, purple trace), under 0Ca^2+^ conditions (0Ca^2+^ + GSK; 20 µM, black trace), and in the presence of the TRPV4 antagonist RN-1734 (RN; 20 µM, 60 min, green trace). The arrow indicates the time of GSK application. For clarity, the baseline of the traces has been adjusted to zero. (**B**) Summary data showing the mean ± SEM of peak [Ca^2+^]_i_ amplitudes in the cells under GSK conditions (purple bar), in 0Ca^2+^ conditions plus GSK (black bar), and in the presence of the TRPV4 antagonist RN-1734 (green bar), expressed in A.U. (**C**) Representative [Ca^2+^]_i_ traces showing the Ca^2+^ response to AA (blue trace) in the absence or presence of RN-1734 (RN; 20 µM, 60 min, pink trace). The arrow indicates the time of AA application. For clarity, the baseline of the traces has been adjusted to zero. (**D**) Summary data showing the mean ± SEM of peak [Ca^2+^]_i_ amplitudes in the absence (blue bar) or presence (pink bar)of RN-1734 (**C**), expressed in A.U. A statistical comparison was performed using the Mann–Whitney test (****, *p* < 0.0001) for panels (**B**,**D**). Numbers inside the bars indicate the number of cells analysed (*n*), as indicated in parentheses for three independent experiments.

The Ca^2+^-permeable TRPV4 channel responds to mechanical and chemical stimuli, including AA and its metabolites [13,20,67,68,69]. TRPV4 has also been implicated in pulmonary fibrosis, where it promotes fibroblast-to-myofibroblast differentiation [70]. In the present study, we first confirmed TRPV4 protein expression in WI-38 cells using Western blot analysis (Figure S1B), and subsequently investigated its involvement by applying the selective TRPV4 agonist GSK-1016790A (GSK; 20 nM), which elicited a robust increase in [Ca^2+^]_i_ (Figure 6A, GSK, purple trace), but not in the absence of extracellular Ca^2+^ (Figure 6A, 0Ca^2+^ + GSK, black trace) (GSK: 0.48 ± 0.045 A.U., *n* = 49 vs. 0Ca^2+^ + GSK: 0.03 ± 0.005 A.U. *n* = 35; ****, *p* < 0.0001; see Figure 6A,B). The GSK-induced increase in Ca^2+^ was reduced by approximately 60% following pre-incubation with RN-1734 (RN; 20 µM, 60 min, green trace), a selective TRPV4 blocker [71] (GSK: 0.48 ± 0.045 A.U., *n* = 49 vs. GSK + RN: 0.19 ± 0.023 A.U. *n* = 43; ****, *p* < 0.0001; see Figure 6A,B). These findings confirm the presence of functional TRPV4 channels in WI-38 cells. Next, we explored the role of TRPV4 in AA-evoked Ca^2+^ signalling. As shown in Figure 6C,D, pre-treatment with RN (20 µM; 60 min) reduced the magnitude of the AA-evoked Ca^2+^ response by approximately 60% (AA: 0.29 ± 0.023 A.U., *n* = 37 vs. AA + RN: 0.12 ± 0.014 A.U., *n* = 42; ****, *p* < 0.0001).

**Figure 7:**
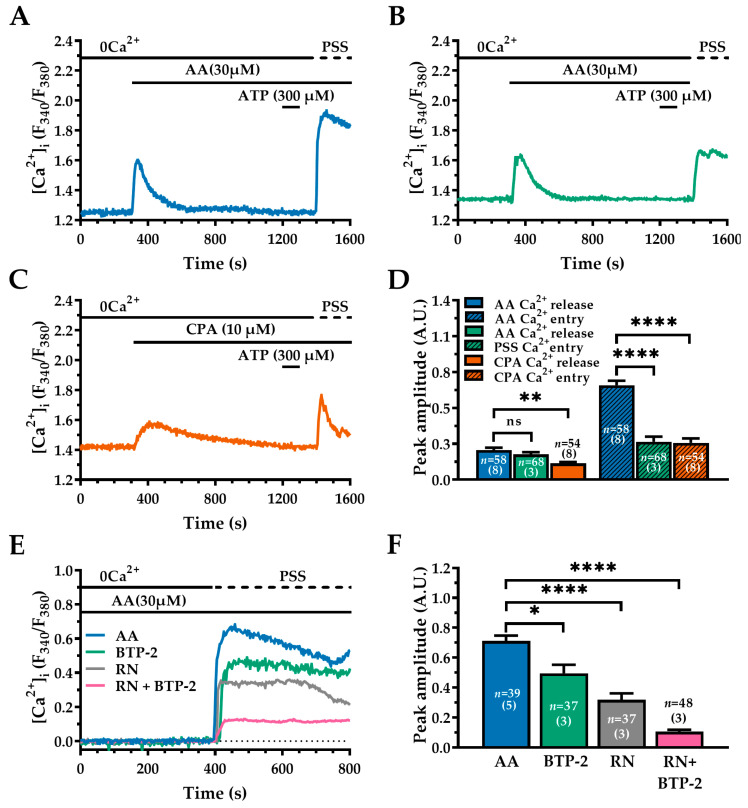
AA-activates Ca^2+^ entry through TRPV4 and SOCE in WI-38 human lung fibroblasts. Representative traces showing [Ca^2+^]_i_ responses recorded in WI-38 cells evoked by AA 30 µM (**A**,**B**) or CPA (**C**) under 0Ca^2+^. Complete ER Ca^2+^ depletion was confirmed by the absence of an intracellular Ca^2+^ response to ATP (300 µM). After ATP washout, extracellular Ca^2+^ was restored by re-addition of PSS, either in the continued presence of AA (PSS + AA, 30 µM, (**A**)), alone (PSS, (**B**)), or in the presence of CPA (PSS + CPA, 10 µM, (**C**)). (**D**) Mean ± SEM of peak Ca^2+^ amplitudes corresponding to Ca^2+^ release under 0Ca^2+^ conditions (**left** panel) and Ca^2+^ entry following Ca^2+^ re-addition (**right** panel) for the conditions showed in (**A**–**C**), expressed in A.U. Statistical analysis was performed using the Kruskal–Wallis test. Significance levels are indicated (ns, not significant; ** *p* < 0.001; **** *p* < 0.0001). (**E**) Representative traces illustrating AA-evoked extracellular Ca^2+^ entry following restoration of extracellular Ca^2+^. The blue trace corresponds to PSS re-addition in the continued presence of AA and represents the control condition. The remaining traces show AA-induced Ca^2+^ entry recorded in the presence of the SOCE inhibitor BTP-2 (20 µM, green trace), the selective TRPV4 inhibitor RN-1734 (RN, 20 µM, grey trace), or the combined treatment (RN + BTP-2; pink trace). For clarity, the baseline of the traces has been adjusted to zero. (**F**) Mean ± SEM of peak Ca^2+^ entry amplitudes under the indicated conditions in (**E**), expressed in A.U. Statistical analysis was performed using the Kruskal–Wallis test (* *p* < 0.05; **** *p* < 0.0001). The *n* value represents the number of cells analysed, and the number of independent experimental replicates is indicated in parentheses.

The evidence that AA-induced intracellular Ca^2+^ mobilisation is supported by IP_3_Rs suggests that the following reduction in the free ER Ca^2+^ concentration can also activate store-operated Ca^2+^ entry (SOCE) [53,55]. Therefore, we first compared AA-induced Ca^2+^ entry with that evoked by CPA, which is a well-known SOCE activator [72]. As shown in Figure 7, under 0Ca^2+^ conditions, both AA (30 µM; Figure 7A, blue and Figure 7B, green trace; and CPA (10 µM; Figure 7C, orange trace) caused a transient increase in [Ca^2+^]_i_, which is consistent with Ca^2+^ release from intracellular stores. Statistical comparisons are shown in Figure 7D (right panel). Both AA and CPA produced complete ER depletion, as evidenced by the absence of any detectable Ca^2+^ response to ATP (300 µM), Figure 7A–C. Re-addition of extracellular Ca^2+^ caused a robust Ca^2+^ entry, which was significantly greater in the presence (Figure 7A, blue trace), rather than in the absence (Figure 7B, green trace), of the agonist: AA: 0.71 ± 0.033 A.U., *n* = 58 vs. PSS: 0.28 ± 0.038 A.U., *n* = 68; ****, *p* < 0.0001 (Figure 7A,B,D, left panel). The same was true when AA-evoked Ca^2+^ entry was compared with CPA (Figure 7C, orange trace): AA: 0.71 ± 0.033 A.U., *n* = 58 vs. CPA: 0.27 ± 0.034 A.U., *n* = 54; ****, *p* < 0.0001 (Figure 7C,D, left panel). These results suggest that AA activates both store-independent Ca^2+^ influx (TRPV4), and store-dependent Ca^2+^ influx pathways.

To further support this hypothesis, we repeated the Ca^2+^ add-back protocol in cells pre-treated with the selective SOCE inhibitor BTP-2 (20 µM) and/or the TRPV4 inhibitor RN (20 µM) [73]. Extracellular Ca^2+^ was reintroduced in the presence of AA (30 µM) to activate TRPV4 channels (see Figure 7E). Inhibiting SOCE with BTP-2 (green trace) produced a modest but significant reduction of AA-induced Ca^2+^ entry (AA: 0.71 ± 0.035 A.U., *n* = 39 vs. BTP-2: 0.49 ± 0.057 A.U., *n* = 37; *, *p* < 0.05; see Figure 7E,F). In contrast, inhibiting TRPV4 with RN-1734 (RN; 20 µM, 60 min, grey trace) reduced the amplitude of AA-induced Ca^2+^ entry by approximately 60% (AA: 0.71 ± 0.035 A.U., *n* = 39 vs. RN: 0.31 ± 0.043 A.U., *n* = 37; ****, *p* < 0.0001; see Figure 7E,F). Notably, combined treatment with RN-1734 and BTP-2 was almost suppressed AA-induced Ca^2+^ entry, reducing the response to near-baseline levels (AA: 0.71 ± 0.035 A.U., *n* = 39 vs. BTP-2 + RN: 0.10 ± 0.012 A.U., *n* = 48; ****, *p* < 0.0001; see Figure 7E,F).

Together, these data demonstrate that AA-induced Ca^2+^ entry in WI-38 cells is mediated primarily by TRPV4 channels, with a smaller contribution from SOCE.

### 2.7. AA Stimulates NO Production in WI-38 Human Lung Fibroblasts

**Figure 8:**
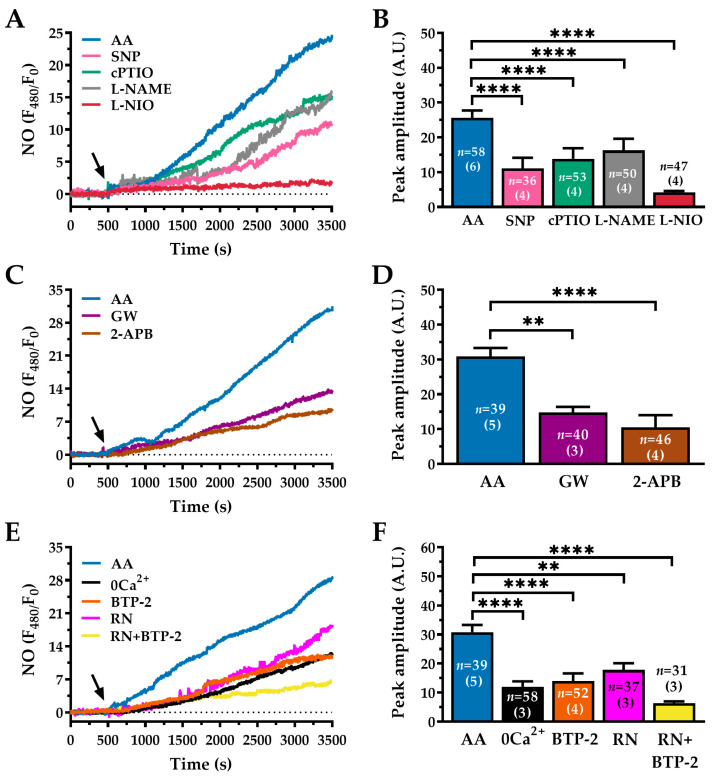
AA stimulates NO production in WI-38 human lung fibroblasts via a Ca^2+^-dependent pathway. (**A**) Representative traces showing NO production, as measured by DAF-FM fluorescence, in response to AA (30 µM, blue). For comparison, traces are shown for the NO donor sodium nitroprusside (SNP; 500 µM, pink), AA stimulation in the presence of the NO scavenger cPTIO (10 µM, green), the non-selective eNOS inhibitor L-NAME (100 µM, 60 min, grey), and the selective eNOS inhibitor L-NIO (50 µM, 60 min, red). The arrow indicates the time of AA (30 µM) stimulation. For clarity, the baseline of the traces has been adjusted to zero. (**B**) Summary bar graph of the peak NO production amplitudes from the experiments shown in (**A**). Data are presented as mean ± SEM, expressed in A.U. Statistical significance was determined by a Kruskal–Wallis test (****, *p* < 0.0001). (**C**) Representative traces showing AA-induced NO production under control conditions (AA; blue) and following the inhibition of key Ca^2+^ signalling components with: GW1100 (10 µM, 10 min, purple) and 2-APB (50 µM, 20 min, brown). The arrow indicates the time of AA (30 µM) stimulation. For clarity, the baseline of the traces has been adjusted to zero. (**D**) Summary bar graph of the peak NO production amplitudes from experiments in (**C**), expressed in A.U. Data are presented as mean ± SEM. Statistical significance was determined by a Kruskal–Wallis test compared to the control (**, *p* < 0.01; ****, *p* < 0.0001). (**E**) Representative traces of AA-induced NO production in the presence of a 0Ca^2+^ solution (black trace); of SOCE inhibition by BTP-2 (20 µM, 20 min, orange trace); and TRPV4 inhibition by RN-1734 (20 µM, 60 min, magenta trace); and under combined inhibition with RN-1734 plus BTP-2 at the same concentrations described above (yellow trace). The arrow indicates the time of AA (30 µM) stimulation. For clarity, the baseline of the traces has been adjusted to zero. (**F**) Summary bar graph of the peak NO production amplitudes under the conditions shown in (**E**) expressed in A.U. Data are presented as mean ± SEM. Statistical significance was determined by a Kruskal–Wallis test compared to the control (**, *p* < 0.01; ****, *p* < 0.0001). The *n* value represents the number of cells analysed, and the number of independent experimental replicates is indicated in parentheses.

An increase in alveolar NO concentration is emerging as a hallmark of IPF [22], but the underlying mechanisms are still unclear. Although it has been demonstrated that AA stimulates NO production in various cell types [13,20,74], it is still unknown whether it does so in human lung fibroblasts. To investigate this, we measured NO production in WI-38 cells using the NO-sensitive fluorescent probe 4-amino-5-methylamino-2′,7′-difluorofluorescein diacetate (DAF-FM, 1 µM, 60 min) [20]. Stimulation with AA (30 µM) triggered a sustained increase in DAF-FM fluorescence, indicating robust NO production (Figure 8A, blue trace). This response was significantly greater in amplitude than that elicited by the exogenous NO donor, sodium nitroprusside (SNP; 500 µM, pink trace) [75] (AA: 25.61 ± 2.122 A.U., *n* = 58 vs. SNP: 11.05 ± 3.107 A.U., *n* = 36; ****, *p* < 0.0001; Figure 8A,B).

The specificity of the AA-induced signal was further confirmed by using pharmacological inhibitors. Pre-incubating WI-38 cells with 2-(4-carboxyphenyl)-4,4,5,5-tetramethylimidazoline-1-oxyl-3-oxide (cPTIO; 10 µM, 60 min, green trace), a NO scavenger [76], reduced AA-induced NO production by approximately 48% (AA: 25.61 ± 2.122 A.U., *n* = 58 vs. cPTIO: 13.85 ± 3.070 A.U., *n* = 53; ****, *p* < 0.0001; Figure 8A,B). Similarly, treatment with the non-selective competitive eNOS inhibitor L-N^G^-nitro-L-arginine methyl ester (L-NAME; 100 µM, 60 min, grey trace) significantly attenuated AA-induced NO release (AA: 25.61 ± 2.122 A.U., *n* = 58 vs. L-NAME: 16.27 ± 3.351 A.U., *n* = 50; ****, *p* < 0.0001; Figure 8A,B). Notably, pre-treatment with the selective eNOS inhibitor L-NIO [77] (50 µM, 60 min, red trace) almost completely abrogated AA-evoked NO production (AA: 25.61 ± 2.122 A.U., *n* = 58 vs. L-NIO: 4.17 ± 0.435 A.U., *n* = 47; ****, *p* < 0.0001; Figure 8A,B).

Since AA increases the [Ca^2+^]_i_ in these cells via the GPR40 receptor, we investigated whether this pathway mediates NO production. GW1100 (GW; 10 µM, 10 min) reduced AA-induced NO generation by approximately 39%, thereby demonstrating the involvement of GPR40 receptors in the NO signal produced by AA (AA: 30.85 ± 2.475 A.U., *n* = 39 vs. GW: 14.83 ± 1.573 A.U., *n* = 40; **, *p* < 0.01; Figure 8C,D). Furthermore, blocking the downstream IP_3_Rs with 2-APB (50 µM, 30 min) caused an even greater reduction in NO release by around 65% (AA: 30.85 ± 2.475 A.U., *n* = 39 vs. 2-APB: 10.58 ± 3.430 A.U., *n* = 46; ****, *p* < 0.0001; Figure 8C,D). This finding confirms that GPR40-dependent ER Ca^2+^ release through IP_3_Rs is critical for this response.

We then examined the contribution of external Ca^2+^ entry to AA-induced NO production. Removal of external Ca^2+^ (0Ca^2+^) reduced the AA-induced NO signal by approximately 60% (AA: 30.85 ± 2.475 A.U., *n* = 39 vs. 0Ca^2+^: 12.01 ± 1.865 A.U., *n* = 58; ****, *p* < 0.0001; Figure 8E,F). This finding indicates that Ca^2+^ influx from the extracellular space substantially contributes to this response. Ca^2+^ imaging revealed that AA-induced Ca^2+^ entry in WI-38 cells is mediated by SOCE and TRPV4 channels. Pharmacological inhibition of SOCE with BTP-2 (20 µM, 20 min) significantly attenuated NO production by around 55% (AA: 30.85 ± 2.475 A.U., *n* = 39 vs. BTP-2: 13.96 ± 2.677 A.U., *n* = 52; ****, *p* < 0.0001; Figure 8E,F). Moreover, inhibition of TRPV4 with RN-1734 (RN; 20 µM, 60 min, magenta trace) reduced the magnitude of the AA-induced NO response by approximately 40% (AA: 30.85 ± 2.475 A.U., *n* = 39 vs. AA + RN: 17 ± 2.303 A.U., *n* = 37; **, *p* < 0.001; Figure 8E,F). Finally, simultaneous inhibition of SOCE and TRPV4 by combined pre-incubation with RN-1734 (RN; 20 µM, 60 min) and BTP-2 (20 µM, 20 min) resulted in a pronounced suppression of the AA-induced NO signal (AA: 30.85 ± 2.475 A.U., *n* = 39 vs. AA + RN + BTP-2: 6.37 ± 0.609 A.U., *n* = 31; ****, *p* < 0.0001; Figure 8E,F). These findings indicate that these two Ca^2+^ entry pathways cooperatively sustain NO production downstream of AA stimulation.

It has long been known that NO may in turn modulate the Ca^2+^ signalling machinery [78,79,80]. To determine whether NO may in turn modulate the Ca^2+^ response to AA, WI-38 cells were pre-incubated for 60 min with the selective eNOS inhibitor L-NIO (10 µM) and the NO scavenger cPTIO (10 µM), before stimulation with AA under both Ca^2+^-containing (PSS) and Ca^2+^-free conditions (0Ca^2+^). As shown in Figure S6A,B, inhibition and scavenging of NO did not significantly alter AA-induced Ca^2+^ signals in the presence of PSS (PSS: 0.31 ± 0.017 A.U., *n* = 90 vs. PSS + cPTIO + L-NIO: 0.33 ± 0.015 A.U., *n* = 86; ns, *p* > 0.05). Similarly, in a Ca^2+^-free buffer, AA elicited a comparable Ca^2+^ signal in both the control group and the treated group (0Ca^2+^: 0.10 ± 0.008 A.U., *n* = 113 vs. 0Ca^2+^ + cPTIO + L-NIO: 0.09 ± 0.004 A.U., *n* = 93; ns, *p* > 0.05, see Figure S6C,D). These findings suggest that NO does not act upstream of Ca^2+^ signaling and does not amplify the Ca^2+^ response to AA.

In conclusion, our data demonstrate that AA-induced NO production in WI-38 cells requires a biphasic Ca^2+^ signal involving the initial release of Ca^2+^ from intracellular stores via GPR40/IP_3_Rs, accompanied by the mobilisation of lysosomal Ca^2+^ through TPC1-2 channels. This is then followed by a sustained influx of Ca^2+^ from the extracellular space through TRPV4 channels and SOCE. Together, these findings support a model in which Ca^2+^-dependent eNOS activation is the main pathway underlying AA-induced NO production in human lung fibroblasts.

### 2.8. AA Stimulates the Production of ROS in WI-38 Human Lung Fibroblasts

**Figure 9:**
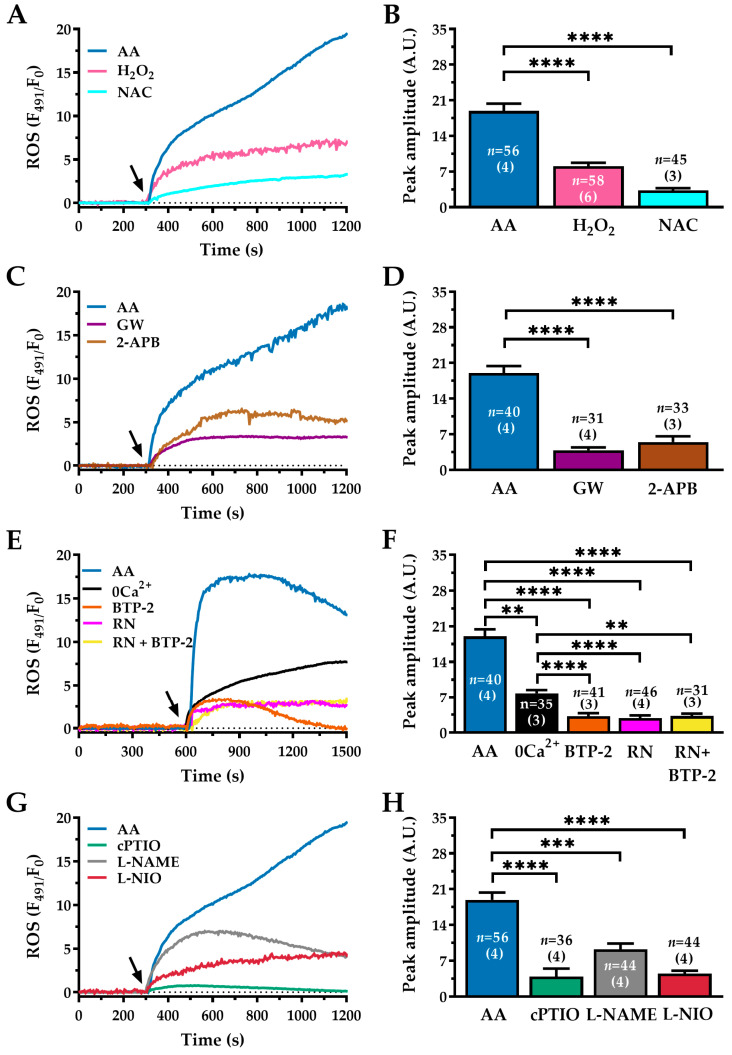
AA-induced ROS generation in WI-38 human lung fibroblasts requires intracellular Ca^2+^ release, Ca^2+^ influx, and NO production. (**A**) Representative traces showing ROS production induced by AA (30 µM, blue) and H_2_O_2_ (100 µM, pink). Pre-treatment with the antioxidant NAC (1 mM, 60 min, cyan). The arrow indicates the time of stimulation. For clarity, the baseline of the traces has been adjusted to zero. (**B**) Quantification of peak ROS fluorescence from (**A**), expressed in A.U. Mean ± SEM. Kruskal–Wallis test; ****, *p* < 0.0001. (**C**) Representative traces of AA-induced ROS production and its modulation by Ca^2+^ signalling inhibitors. Cells were stimulated with AA (30 µM) in PSS (blue trace), following pretreatment with the GPR40 antagonist GW1100 (GW; 10 µM, 10 min, purple), the IP_3_Rs inhibitor 2-APB (50 µM; 20 min, brown). The arrow indicates the time of stimulation. For clarity, the baseline of the traces has been adjusted to zero. (**D**) Quantification of peak ROS fluorescence intensities from (**C**), expressed in A.U. Data are the mean ± SEM of ROS production peak amplitudes. The statistical test used was the Kruskal–Wallis test: ****, *p* < 0.0001. (**E**) Representative traces of AA-induced ROS production under Ca^2+^-free solution (0Ca^2+^; black) or in the presence of the SOCE inhibitor BTP-2 (20 µM, 20 min, orange) or the TRPV4 inhibitor RN-1734 (20 µM, 60 min, magenta), or the combined treatment (RN + BTP-2; yellow trace). The arrow indicates the time of AA (30 µM) stimulation. For clarity, the baseline of the traces has been adjusted to zero. (**F**) Quantification of peak ROS fluorescence intensities from (**E**). Data are the mean ± SEM of the peak ROS production amplitudes, expressed in A.U. The statistical test used was the Kruskal–Wallis test: **, *p* < 0.01; ****, *p* < 0.0001. (**G**) A representative trace showing the effect of NO pathway inhibition on AA-induced ROS production. Signals were recorded in response to AA (30 µM) under control conditions (blue), after NO scavenging with cPTIO (10 µM, 60 min, green), followed by non-selective NOS inhibition with L-NAME (100 µM, 60 min, grey), and subsequently by selective eNOS inhibition with L-NIO (20 µM, 60 min, red). The arrow indicates the time of AA (30 µM) stimulation. For clarity, the baseline of the traces has been adjusted to zero. (**H**) Quantification of peak ROS fluorescence intensities from (**G**). The data are the mean ± SEM of the peak ROS production amplitudes, expressed in A.U. The statistical test used was the Kruskal–Wallis test: ***, *p* < 0.001; ****, *p* < 0.0001. For all panels, the *n* value represents the number of cells analysed, and the number of independent experimental replicates is indicated in parentheses.

Having established that AA stimulates Ca^2+^-dependent NO production, we investigated whether it also induces ROS generation, which plays a key role in the pathophysiology of IPF. Using the ROS-sensitive probe 2′,7′-dichlorodihydrofluorescein diacetate (H_2_DCF-DA), we found that AA (30 µM) triggered a rapid and sustained increase in fluorescence, indicating robust ROS generation (Figure 9A, blue trace). This response was approximately 133% greater than that elicited by the positive control (hydrogen peroxide, H_2_O_2_; 100 µM, pink trace) (AA: 18.91 ± 1.435 A.U., *n* = 56 vs. H_2_O_2_: 8.03 ± 0.706 A.U., *n* = 58; ****, *p* < 0.0001; Figure 9A,B). The specificity of this signal was confirmed by pretreatment with the antioxidant N-acetylcysteine (NAC; 1 mM, cyan trace), which acts as both a direct antioxidant and a glutathione precursor [81]. NAC reduced AA-induced ROS production by around 82% (AA: 18.91 ± 1.435 A.U., *n* = 56 vs. NAC: 3.3 ± 0.431 A.U., *n* = 45; ****, *p* < 0.0001; Figure 9A,B).

Given the well-established crosstalk between Ca^2+^ and ROS signalling, we investigated the mechanism underlying AA-induced ROS generation. Inhibiting the GPR40 receptor with GW1100 (GW; 10 µM) reduced ROS production by approximately 79.5%, indicating that this receptor activates the pathway (AA: 19.03 ± 1.358 A.U., *n* = 40 vs. GW: 3.86 ± 0.555 A.U., *n* = 31; ****, *p* < 0.0001; Figure 9C,D). Similarly, blocking downstream IP_3_Rs-mediated Ca^2+^ release with 2-APB (50 µM) significantly reduced ROS levels by approximately 72% (AA: 19.03 ± 1.358 A.U., *n* = 40 vs. 2-APB: 5.46 ± 1.142 A.U., *n* = 33; ****, *p* < 0.0001; Figure 9C,D). These data confirm that AA-induced ROS generation is directly driven by the GPR40–PLCβ–IP_3_Rs signalling axis.

We then assessed the contribution of extracellular Ca^2+^ influx to AA-induced ROS production. Stimulation with AA under 0Ca^2+^ conditions reduced ROS generation by around 60% (AA: 19.03 ± 1.358 A.U., *n* = 40 vs. 0Ca^2+^: 7.84 ± 0.627 A.U., *n* = 35; **, *p* < 0.01; Figure 9E,F). Similarly, the selective inhibition of SOCE with BTP-2 (20 µM) decreased AA-induced ROS production (AA: 19.03 ± 1.358 A.U., *n* = 40 vs. BTP-2: 3.20 ± 0.608 A.U., *n* = 41; ****, *p* < 0.0001; Figure 9E,F); a similar effect was obtained by blocking TRPV4 channels with RN reduced AA-induced ROS production (AA: 19.03 ± 1.358 A.U., *n* = 40 vs. RN: 2.84 ± 0.529 A.U., *n* = 46; ****, *p* < 0.0001; Figure 9E,F). Finally, simultaneous inhibition of SOCE and TRPV4 by combined pre-incubation with RN-1734 (RN; 20 µM, 60 min) and BTP-2 (20 µM, 20 min) also suppressed the AA-induced ROS signal (AA: 17.03 ± 1.358 A.U., *n* = 40 vs. AA + RN + BTP-2: 3.24 ± 0.478 A.U., *n* = 31; ****, *p* < 0.0001; Figure 9E,F). Together, these results suggest that influx of Ca^2+^ through both SOCE and TRPV4 channels is a critical requirement for full ROS generation in response to AA. Recent evidence indicates that NO release can stimulate ROS generation [25,26]. To determine whether NO contributes to AA-induced ROS signalling, NO was scavenged using cPTIO (10 µM). This intervention reduced AA-induced ROS production by approximately 80%, identifying NO as a major driver of ROS production (AA: 18.91 ± 1.435 A.U., *n* = 56 vs. cPTIO: 3.89 ± 1.559 A.U., *n* = 36; ****, *p* < 0.0001; Figure 9G,H). In addition, eNOS activity was inhibited using L-NAME (100 µM), and the more selective eNOS inhibitor L-NIO (50 µM). Inhibition of eNOS reduced AA-induced ROS production by 51% in the presence of L-NAME (AA: 18.91 ± 1.435 A.U., *n* = 56 vs. L-NAME: 9.22 ± 1.133 A.U., *n* = 44; ***, *p* < 0.001; Figure 9G,H), and 76% in the presence of L-NIO (AA: 18.91 ± 1.435 A.U., *n* = 56 vs. L-NIO: 4.46 ± 0.580 A.U., *n* = 44; ****, *p* < 0.0001; Figure 9G,9H), respectively.

These findings suggest that NO production supports ROS generation. Nicotinamide adenine dinucleotide phosphate (NADPH) oxidase 2 (NOX2) is the only recognized NO-sensitive NOX isoform [25,26]. Pharmacological inhibition of NOX2 with GSK2795039 (30 µM, for 2 h) significantly attenuated AA-induced ROS production, thereby supporting its involvement as a major enzymatic source of ROS in this context (AA: 19.02 ± 1.569 A.U., *n* = 82 vs. GSK: 3.54 ± 0.361 A.U., *n* = 79; ****, *p* < 0.0001; see Figure S7A,B).

To assess whether oxidative signalling may in turn promote NO release, we measured AA-induced NO production in WI-38 cells pretreated with NAC (1 mM, 60 min). NAC did not significantly alter AA-induced NO production, as measured by DAF fluorescence (AA: 25.61 ± 2.122 A.U., *n* = 58 vs. NAC: 23.62 ± 3.537 A.U., *n* = 49; ns, *p* > 0.05; see Figure S8A,B). This finding suggests that NO generation occurs upstream of ROS signalling and is not directly affected by antioxidant treatment. This supports the view that ROS production is a secondary event that occurs after NO signalling.

Taken together, these results support a model in which AA-induced ROS generation requires Ca^2+^-dependent NO production, followed by NOX2 activation and subsequent ROS generation in WI-38 cells.

## 3. Discussion

This study defines a Ca^2+^–NO–ROS signalling axis activated by AA in human WI-38 lung fibroblasts, providing mechanistic insights into lipid-driven fibroblast dysfunction associated with pulmonary fibrosis. Using a combination of Ca^2+^, NO, and ROS imaging alongside targeted pharmacological interventions, we demonstrate that unmetabolized AA induces a two-phase Ca^2+^ response. This involves the release of Ca^2+^ from the ER and lysosomes, followed by sustained Ca^2+^ influx, which is predominantly mediated by TRPV4 channels and, to a lesser extent, by SOCE. This Ca^2+^ signal is functionally coupled to NO synthesis and ROS generation, revealing a tightly interconnected redox signalling axis.

### 3.1. GPR40–PLC–IP_3_Rs Signalling Underlies the Initial Ca^2+^ Release

AA evoked a concentration-dependent increase in [Ca^2+^]_i_ with an *EC*_50_ in the low micromolar range. This concentration aligns with the elevated levels of AA and its metabolites found in inflamed pulmonary microenvironments, highlighting the pathophysiological relevance of AA signalling in chronic inflammatory and fibrotic conditions [82,83]. Furthermore, the non-metabolizable analogue ETYA was able to reproduce the AA-evoked Ca^2+^ response in WI-38 cells. This observation supports the conclusion that AA exerts a direct signalling role rather than an indirect effect mediated by downstream eicosanoids or non-specific membrane perturbation.

Pharmacological manipulation identifies GPR40 as a major upstream sensor of AA in WI-38 human lung fibroblasts. GPR40 primarily couples to Gq proteins, activating PLCβ and generating IP_3_ and DAG. IP_3_ then binds to IP_3_Rs on the ER, triggering Ca^2+^ release [84]. Pharmacological blockade of GPR40, PLCβ, or IP_3_Rs almost abolished ER Ca^2+^ release, whereas PTX had no effect. This indicates that GPR40 is mainly coupled to a protein Gq/PLC/IP_3_Rs pathway. These findings are consistent with reports in vascular endothelial cells and dermal fibroblasts [13,20] and extend them to lung fibroblasts, which are central to fibrotic remodelling. Although the RyR_1_ isoform is expressed in WI-38 cells [53], the lack of a Ca^2+^ response to caffeine suggests that the IP_3_Rs represent a major ER Ca^2+^ release mechanism engaged by AA.

### 3.2. The ER and Lysosomes Ca^2+^ Stores Cooperate in Ca^2+^ Release

Beyond the ER, our data reveal a substantial contribution from lysosomal Ca^2+^ stores to the AA-evoked Ca^2+^ signal. When ER Ca^2+^ uptake was inhibited by CPA, AA still induced a measurable, albeit reduced, increase in cytosolic Ca^2+^, suggesting the involvement of non-ER Ca^2+^ sources. Pharmacological evidence implicates lysosomes as a major contributor, as AA-induced intracellular Ca^2+^ release was markedly attenuated by GPN, which selectively disrupts the cytosolic Ca^2+^ pool [85]. TPCs represent the major pathway for agonist-induced lysosomal Ca^2+^ mobilisation [86,87], selective inhibition of NAADP signalling with Trans-Ned 19 almost completely abolished the AA-induced Ca^2+^ response, underscoring the functional relevance of TPCs in Ca^2+^ mobilisation from acidic stores [63,88].

The participation of lysosomal Ca^2+^ stores is consistent with emerging models proposing functional communication between the ER and lysosomes at membrane contact sites, where localised Ca^2+^ microdomains facilitate signal amplification and inter-organelle coordination. Such spatial coupling provides a plausible mechanism by which relatively small, localised lysosomal Ca^2+^ release events are converted into robust and sustained global cytosolic Ca^2+^ signals [89]. In line with this model, selective disruption of lysosomal using GPN markedly attenuated the AA-induced Ca^2+^ response, leaving only a minor residual component attributable to ER Ca^2+^ release. These findings suggest that lysosomal Ca^2+^ mobilisation acts upstream of, and is required for, efficient ER-dependent Ca^2+^ release [90].

### 3.3. Sustained Ca^2+^ Influx: TRPV4 and SOCE

The sustained plateau phase observed following AA stimulation clearly depends on the entry of extracellular Ca^2+^. In the absence of extracellular Ca^2+^ (0 Ca^2+^), AA induced a transient increase in [Ca^2+^]_i_ that lacked the plateau phase, while the re-addition of Ca^2+^ fully restored it. Agonist-induced Ca^2+^ entry in fibroblasts may be mediated by SOCE and/or ligand-gated cation channels [91]. In addition, AA-induced Ca^2+^ entry can be mediated by arachidonate-regulated Ca^2+^ (ARC) channels [92].

SOCE is triggered by the ER Ca^2+^ sensors, stromal interaction molecule (STIM1) and STIM2. Upon store depletion, they interact with the Ca^2+^-selective Orai channels in the plasma membrane to form Ca^2+^-release-activated Ca^2+^ (CRAC) channels [93,94]. This mechanism allows sustained Ca^2+^ influx, promoting store refill and maintenance of the cytosolic Ca^2+^ signal. SOCE represents the main pathway that sustains agonist-evoked Ca^2+^ signals in various fibroblast populations, including pulmonary fibroblasts [95,96,97]. Consistent with this, our previous work demonstrated the predominant expression of STIM2 and Orai3 in WI-38 human foetal lung fibroblasts [53]. The functional relevance of SOCE in these cells was further supported by studies showing that pharmacological inhibition of SOCE prevents surfactant-induced apoptosis and significantly reduces α1(I) procollagen expression [55]. Together, these findings support the concept that SOCE plays a central role in regulating survival and fibro genic signalling in human lung fibroblasts. To dissect the contribution of store-operated vs. agonists-dependent channels, we exploited the Ca^2+^ add-back protocol in the presence and absence of AA when Ca^2+^ was reintroduced into the extracellular solution [98,99,100]. We found that Ca^2+^ entry occurred both in the absence and in the presence of the agonist at the time of Ca^2+^ re-addition. This observation indicates that the previous depletion of the ER Ca^2+^ store under 0Ca^2+^ conditions was able to induce SOCE. Nevertheless, the amplitude of Ca^2+^ influx was significantly larger in the presence of AA, thereby suggesting that agonist-dependent channels contribute to and play a major role in Ca^2+^ entry [98,99]. ARC channels are mediated by the interaction among STIM1, Orai1 and Orai3 [92]. As mentioned above, STIM1 and Orai1 are not expressed in WI-38 lung fibroblasts [53]; accordingly, ARC channels cannot contribute to AA-induced Ca^2+^ entry. Therefore, we hypothesized that TRPV4 accounted for the agonist-dependent component of AA-induced Ca^2+^ entry. First, we found that TRPV4 was expressed and functional in WI-38 lung fibroblasts. Second, the Ca^2+^ add-back protocol further revealed that the combined application of BTP-2 and RN, which respectively inhibit SOCE and TRPV4, suppressed AA-induced Ca^2+^ entry. Third, the inhibitory effect of RN was significantly stronger than that of BTP-2.

Our pharmacological data indicate that TRPV4 is the predominant pathway mediating AA-evoked Ca^2+^ entry, while SOCE makes a smaller yet significant contribution to the sustained Ca^2+^ influx. This dual mechanism for Ca^2+^ influx likely enables robust and flexible Ca^2+^ signalling in response to lipid-derived inflammatory stimuli. Notably, the expression of TRPV4 in lung fibroblasts has been associated with profibrotic phenotypes and mechanochemical signal transduction linked to the extracellular matrix stiffness [101].

In contrast, the non-specific VOCCs blocker Ni^2+^ did not significantly alter the AA-induced Ca^2+^ response. Consistently, membrane depolarisation with high extracellular K^+^ failed to elicit a detectable Ca^2+^ signal in WI-38 cells, suggesting that VOCCs do not contribute to AA-evoked Ca^2+^ entry.

Unexpectedly, the L-type Ca^2+^ channel blocker nifedipine increase basal [Ca^2+^]_i_ when applied alone. Similar effects have been reported in other cell types and have been attributed to the activation of the Ca^2+^-sensing receptor [102,103,104,105]. A comparable lack of VOCC involvement has been described in porcine aortic endothelial cells, which also do not express functional VOCCs. Collectively, these findings suggest that nifedipine may activate alternative, VOCC-independent signalling pathways in diverse non-excitable cell types [106,107].

### 3.4. AA-Induced NO and Redox Signalling: A Unidirectional Ca^2+^–NO–ROS Axis

NO and oxidative stress have long been known to contribute to IPF progression [29,30,31,32,33]. An intimate relationship exists among Ca^2+^ signalling, NO release and oxidative stress. Consistently, an increase in [Ca^2+^]_i_ can induce both NO [108] and ROS production [31], as well as a NO burst can induce oxidative stress [25,26]. By using selective fluorescent probes, we found that AA elicited the production of both NO and ROS. Pharmacological inhibition of GPR40, IP_3_Rs, TRPV4 channels or SOCE significantly attenuated these responses, indicating that the Ca^2+^ rise represents the initial step in AA-induced NO and redox signalling. NO synthesis is mediated by the NO synthase (NOS) family, which comprises the Ca^2+^-dependent nNOS and eNOS isoforms, whereas the inducible NOS (iNOS) is predominantly induced under inflammatory conditions and is Ca^2+^-independent [109]. In lung myofibroblasts, both iNOS and eNOS have been described, with eNOS representing the dominant isoform under basal conditions [110]. Therefore, AA-induced NO production in WI-38 lung fibroblasts is mainly driven by eNOS, as shown in vascular endothelial cells [20,111]. Notably, NO production was not required to sustain the Ca^2+^ response to AA, thereby ruling out the possibility that NO exerts a positive feedback on the Ca^2^ signalling machinery. Strikingly, disrupting NO signalling with L-NAME, L-NIO and c-PTIO also attenuated AA-induced ROS production. This observation suggests that NO production is required to stimulate ROS generation. Consistent with this hypothesis, NO has long been known to activate the soluble guanylyl cyclase/cyclic guanosine monophosphate (cGMP)/protein kinase G (PKG) signalling pathway. PKG, in turn, stimulates nicotinamide adenine dinucleotide phosphate (NADPH) oxidase 2 (NOX2), which is expressed in lung fibroblasts [112,113] and drives ROS production [25,26]. The main ROS generated by NOX2 are O_2_•^−^ and hydrogen peroxide [114].The virtual suppression of AA-induced ROS production by c-PTIO, which scavenges NO, suggests that oxidative stress is mainly driven by NO rather than by the Ca^2+^-dependent NOX5 [115]. Therefore, NOX2 is the major candidate to sustain AA-induced NO release, although weak NO production occurs following inhibition of NO-signalling. This finding suggests that NOX5 may also play a minor role. Overall, these findings indicate that AA stimulates ROS generation through the Ca^2+^-dependent production of NO. Notably, IPF patients exhibit significantly elevated alveolar NO levels, which correlate with severity disease [22,116,117]. Moreover, excessive production of NO correlates with the burden of oxidative/nitrosative stress and has been associated with aberrant fibrotic remodelling in mouse models of IPF [118]. The evidence that Ca^2+^-dependent NO release is essential for ROS generation in human lung fibroblasts highlights a novel signalling pathway with the potential to drive IPF progression. While it has long been proposed that NO and ROS are simultaneously produced by two parallel signalling pathways [119], these findings demonstrate that, in lung fibroblasts, they can be integrated within the same biochemical cascade. According to this model, as ROS are produced, they react with NO to form reactive nitrosative species, including ONOO^−^, thereby favouring IPF progression. Notably, dysregulated AA metabolism can exacerbate lung injury and fibrosis via eicosanoid mobilisation [14,120,121]. Herein, we further demonstrate that AA also elicits nitrosative and oxidative stress through an increase in [Ca^2+^]_i_ in lung fibroblasts, adding a further layer of complexity to the strong association between aberrant lipid signalling and IPF pathogenesis [120,122]. A successful pharmacological approach to treat IPF is still lacking as both antioxidants, such as NAC, or antifibrotic drugs have failed to achieve a significant therapeutic effect.

AA also induced an immediate increase in [Ca^2+^]_i_ and in NO production. These observations are consistent with the signalling sequence proposed in the present study, whereby an increase in [Ca^2+^]_i_ leads to eNOS activation, NO generation, and subsequent ROS production. The incomplete inhibition of NO production and H_2_DCF-DA oxidation by antagonists of Ca^2+^ entry is not unexpected, as AA is still able to mobilize Ca^2+^ from intracellular neutral and acidic Ca^2+^ stores. Accordingly, AA-induced NO production and H_2_DCF-DA oxidation were significantly reduced in the absence of extracellular Ca^2+^ and were strongly inhibited by GW1100, a GPR40 antagonist, as well as by 2-APB, which blocks IP_3_Rs. This finding may be explained by hypothesizing that the simultaneous pre-incubation with BTP-2 and RN could have somehow depleted the intracellular Ca^2+^ stores, thereby reducing AA-induced Ca^2+^ release and eNOS activation, as shown in human cerebrovascular endothelial cells [123]. Alternatively, we could speculate that the co-activation of SOCE and TRPV4 is necessary or AA to bring about a measurable elevation in DAF-FM fluorescence under 0Ca^2+^ conditions. It is conceivable that eNOS lies in close proximity with different Ca^2+^-permeable conductances, i.e., InsP_3_Rs, Orai3 and TRPV4, and that their combined recruitment is needed to elevate NO levels in WI-38 cells, as shown in human endothelial colony forming cells [13].

The evidence that AA elicits intracellular Ca^2+^ signals to induce the production of NO and ROS highlights novel signalling pathways that could be targeted for therapeutic purposes in IPF. Consistent with this hypothesis, TPCs, TRPV4 and SOCE are emerging are promising molecular candidates to treat several pathologies [124,125,126,127]. Future work should assess whether targeting the Ca^2+^/NO/ROS feedback mechanism mitigates IPF progression in animal models of fibrotic remodelling.

## 4. Materials and Methods

### 4.1. Cell Culture

Human foetal human lung fibroblasts (WI-38; CCL-75™) were obtained from the American Type Culture Collection (ATCC^®^, Manassas, VA, USA). The cells were cultured in Minimum Essential Medium (MEM; Biowest, Nuaillé, France), supplemented with 10% foetal bovine serum (FBS; Biowest, Nuaillé, France) and 1% antibiotic-antimycotic solution (containing 100 U/mL penicillin, 100 µg/mL streptomycin, and 0.25 µg/mL amphotericin B; Sigma-Aldrich, St. Louis, MO, USA). The cultures were maintained in T-25 culture flasks with ventilated caps at 37 °C in a humidified incubator with 5% CO_2_. The culture medium was replaced every 72 h. Upon reaching approximately 80% confluence, the cells were harvested with 0.25% Trypsin-EDTA (Gibco, Grand Island, NY, USA) and seeded onto sterile glass coverslips in 35 mm Petri dishes 24 h before the experiments. To ensure consistency, only fibroblasts between passages 5 and 14 were used.

### 4.2. Physiological Solutions

PSS was prepared with the following composition (in mM): 150 NaCl, 6 KCl, 1.5 CaCl_2_, 1 MgCl_2_, 10 glucose, and 10 HEPES. This solution was used both for loading cells with Ca^2+^, NO, and ROS-sensitive fluorescent dyes, and to maintain cells during live-cell imaging experiments. For the Ca^2+^-free extracellular solution (0Ca^2+^), CaCl_2_ was omitted and replaced with 2 mM NaCl; 0.5 mM EGTA was added to chelate residual Ca^2+^. All solutions were titrated to pH 7.4 using NaOH, and their osmolarity was verified (300–310 mOsm/L) using a vapor pressure osmometer (Wescor 5500, Logan, UT, USA).

### 4.3. Image Acquisition and Fluorescence Measurements

#### 4.3.1. Ca^2+^ Imaging Protocol

Intracellular Ca^2+^ imaging was performed as previously described [53], Briefly, WI-38 human lung fibroblasts grown on sterile glass coverslips, were loaded with 3 µM Fura-2 acetoxymethyl ester (Fura-2/AM; Invitrogen, #F1201) in PSS at a controlled temperature of 23 ± 1 °C for 30 min. After incubation, cells were washed with dye-free PSS for 30 min under the same temperature conditions. Coverslips were mounted at the bottom of a Petri dish using a drop of silicone and placed on the stage of a vertical epifluorescence microscope (Axiolab, Carl Zeiss, Oberkochen, Germany), equipped with a 50 W mercury lamp (HBO50; OSRAM; Augsburg, Germany). Cells were visualized using a 40× Zeiss Achroplan water immersion objective with a working distance of 2.0 mm and a numerical aperture of 0.9 (Carl Zeiss Microscopy, White Plains, NY, USA). WI-38 cells were excited alternately at wavelengths of 340 and 380 nm using a motorized filter wheel (Lambda 10, Sutter Instrument, Novato, CA, USA). Fluorescence emission was collected at 510 nm using an Extended-ISIS CCD camera (Photonic Science, Millham, UK). A neutral density filter (optical density = 1.0) was paired with the 380 nm filter to approach the light intensity of the 340 nm wavelength. An aperture diaphragm was used to enhance contrast. Custom software, operating in the LINUX environment, was used to drive the camera and the filter wheel. Acquired images were stored on the hard disk and analysed off-line using ImageJ software Version 1.54s (National Institutes of Health, MD, USA, https://imagej.net/ij/ (accessed on 5 March 2020). Individual cells were carefully selected by drawing manually region of interest (ROI) ensuring no overlap with adjacent cells, each ROI was identified by a number. The [Ca^2+^]_i_ was monitored by measuring, for each ROI, the ratio of the mean fluorescence emitted at 510 nm when exciting alternatively at 340 and 380 nm (hereafter termed “Ratio (F_340_/F_380_)”). An increase in [Ca^2+^]_i_ causes an increase in the Ratio (F_340_/F_380_). Ratio measurements were performed and plotted every 3 s, with ratio values expressed in arbitrary units (A.U.).

#### 4.3.2. NO Measurement Protocol

NO production was assessed by incubating WI-38 cells in PSS containing 1 µM of 4-amino-5-methylamino-2′,7′-difluorofluorescein diacetate (DAF-FM; Molecular Probes, #23842) for 60 min at 23 ± 1 °C. Following incubation, cells were washed with dye-free PSS for an additional 60 min at the same temperature. Imaging was conducted by using the same setup described for Ca^2+^ recordings but with a different filter set, excitation at 480 nm and emission collected at 535 nm [123]. Images were acquired every 5 s. ROIs were manually defined in ImageJ, and the mean fluorescence intensity (F_535_/F_0_) was calculated for each ROI and plotted over time. Exogenous L-arginine was not added to the perfusate. This is because the Km of L-arginine for endothelial nitric oxide synthase (eNOS) is approximately 2.9 µM [128], whereas intracellular L-arginine concentrations typically range from 0.8 to 2.0 mM [129]. Therefore, endogenous intracellular L-arginine levels are generally sufficient to maintain eNOS in a substrate-saturated state and support NO production.

#### 4.3.3. ROS Measurement Protocol

ROS production was measured by incubating WI-38 cells in PSS containing 5 µM 2′,7′-dichlorodihydrofluorescein diacetate (H_2_DCF-DA; Sigma-Aldrich, St. Louis, MO, USA) which is hydrolysed by intracellular esterases to yield DCFH. Upon interaction with ROS, DCFH is oxidized to DCF, resulting in increased fluorescence [81]. H_2_DCF-DA was incubated for 30 min in PSS, as described in [81]. Excess dye was removed by washing with PSS. Cells were imaged using the same equipment and acquisition settings described for Ca^2+^ recordings, with excitation and emission filters set at 490 nm and 520 nm, respectively. Images were acquired every 5 s, and the mean fluorescence intensity (F_520_/F_0_) was measured for each ROI using ImageJ and plotted over time.

### 4.4. Drugs Application

The bathing medium (3 mL), sufficient to fully submerge the coverslip, was carefully aspirated using a suction pump and replaced with the desired drug solution. The medium could be substituted quickly without producing artifacts in the fluorescence signal trace because a small meniscus of liquid remained between the tip of the objective and the WI-38 cells. The solution removal and replacement cycle was repeated three times to ensure complete substitution of the previous medium.

### 4.5. Western Blot

Cells were maintained under the experimental conditions described above and harvested at 80% confluency. For protein extraction, culture plates were placed on ice and cells were washed twice with ice-cold PBS to remove residual medium. Cells were then scraped in RIPA lysis buffer (Pierce^®^ RIPA Buffer, Thermo Fisher Scientific, Waltham, MA, USA) supplemented with protease inhibitor cocktail (Halt™ Protease Inhibitor Cocktail, 1:100; Thermo Fisher Scientific, Waltham, MA, USA). Lysates were vortexed briefly, incubated on ice for 10 min, and subsequently clarified by centrifugation at 13,000× g for 15 min at 4 °C. Total protein concentration was quantified using a Bicinchoninic Acid (BCA) assay kit (Merck KGaA, Darmstadt, Germany), according to the manufacturer’s instructions. Equal amounts of proteins (30 μg) were mixed with SDS sample buffer, denatured for 5 min at 95 °C, and resolved on 4–15% Mini-PROTEAN TGX Precast Protein Gels (Bio-Rad, Hercules, CA, USA). Proteins were then electrotransferred onto PVDF membranes (Trans-Blot Turbo Transfer Pack, Bio-Rad, Hercules, CA, USA) using the Trans-Blot Turbo Transfer System (Bio-Rad, Hercules, CA, USA). Membranes were blocked for 1 h at room temperature in TBST (20 mM Tris, 150 mM NaCl, 0.1% Tween 20, pH 7.6) containing 5% BSA and subsequently incubated overnight at 4 °C under gentle agitation with the following primary antibodies: Anti-GPR40 Polyclonal Antibody (#BS-13535R,1:300, in TBST 5% BSA 0.02% sodium azide; Bioss Inc., Woburn, MA, USA) and Anti-TRPV4 Antibody (#ab191580, 1:500, in TBST 5% BSA 0.02% sodium azide; Abcam, Cambridge, UK). After washing in TBST, membranes were incubated with the appropriate HRP-conjugated secondary antibody (1:10,000, in TBST 5% BSA; Thermo Fisher Scientific, Waltham, MA, USA). Protein bands were visualized by chemiluminescence.

### 4.6. Chemicals

Arachidonic acid was obtained from (Sigma-Aldrich, St. Louis, MO, USA); catalog number 10931). Arachidonic acid stock solutions were prepared in absolute ethanol and stored in small aliquots at −20 °C in light-protected tubes to minimize oxidation and avoid repeated freeze–thaw cycles. For each experiment, fresh working solutions were prepared immediately before use by diluting the stock solution into the appropriate physiological solution. The final concentration of ethanol in the experimental medium did not exceed 0.1%, a concentration that had no detectable effect on the measured parameters.

Palmitoleic acid, Trans Ned-19, GPN, and RN-1734 were obtained from ChemCruz Biochemical (Dallas, TX, USA). U-73343, cPTIO and GSK2795039 were sourced from Cayman Chemical (Ann Arbor, MI, USA), while GW1100 was purchased from Merck (Darmstadt, Germany). 2-APB was acquired from Calbiochem (La Jolla, CA, USA). The fluorescent indicators DAF-FM and FURA 2-AM were purchased from Molecular Probes (Europe BV, Leiden, The Netherlands). All other chemical reagents were obtained from Sigma Aldrich.

### 4.7. Data Analysis

Fluorescence traces were analysed using Clampfit 11.3 software (Molecular Devices, San Jose, CA, USA). For Ca^2+^ signal quantification, the peak response amplitude evoked by AA was calculated as the difference between the peak fluorescence ratio (F_340_/F_380_) and the mean baseline ratio (averaged over the 50 s pre-stimulation period).

Dose–response relationship was fitted using the following equation:

(1) $$Y=\frac{100}{1+\frac{{EC}_{50}}{\left[AA\right]}}$$

where *Y* represents the Ca^2+^ response amplitude, [*AA*] is the AA concentration, and *EC*_50_ denotes the half-maximal effective concentration.

NO and ROS signals were analysed by calculating the difference between the peak fluorescence value during post-stimulation periods (3000 s for NO, 1000 s for ROS) and the mean baseline fluorescence (50 s pre-stimulation).

All experiments were conducted at 23 ± 1 °C using cells from at least three independent passages. To control for daily variability, standardized control experiments were conducted in parallel with each experimental protocol. Statistical comparisons were consistently made against same-day controls to minimize inter-day variation in cell responsiveness and experimental conditions.

Statistical analyses and graph generation were performed using GraphPad Prism 9.0 software (GraphPad, San Diego, CA, USA). Results are presented as mean ± standard error of the mean (SEM). Normality was verified for each experimental protocol using the Shapiro–Wilk test. Parametric data were analysed with unpaired Student’s *t* test (two groups) or one-way ANOVA with Dunnett’s post hoc test (multiple comparisons). Non-parametric data were evaluated using Wilcoxon test and Friedman test or Mann–Whitney *U* test (two-groups) or Kruskal–Wallis test (multiple groups). Statistical significance was set at *p* < 0.05 for all analyses. The number of cells analysed for each condition is indicated in the corresponding bar histograms (*n* = total cells analysed, with the number in parentheses indicating biological replicates).

## 5. Conclusions

In summary, AA orchestrates a multifaceted signalling network in human lung fibroblasts characterized by: (i) GPR40-mediated PLC–IP_3_Rs activation; (ii) coordinated ER-lysosome Ca^2+^ release; (iii) sustained Ca^2+^ influx via SOCE and TRPV4 channels; and (iv) downstream activation of Ca^2+^-dependent NO and ROS pathways (see Figure S9). These cascades converge to establish a feed-forward cycle of oxidative and nitrosative stress that may drive fibroblast activation and fibrosis progression. The Ca^2+^-dependent activation of eNOS represents the principal mechanism driving NO synthesis, which in turn triggers NOX2-dependent ROS generation. The delineation of this Ca^2+^–NO–ROS axis provides a mechanistic framework The Ca^2+^-dependent activation of eNOS represents the principal mechanism driving NO synthesis, which in turn triggers NOX2-dependent ROS generation.

Future studies should investigate how chronic exposure to AA or its metabolites modifies gene expression profiles of extracellular matrix components and explore whether pharmacological modulation of GPR40, TPCs, or SOCE components can reverse established fibrosis in vivo. Given the growing evidence that metabolic reprogramming shapes fibroblast phenotypes, targeting lipid-dependent Ca^2+^ signalling represents a promising strategy for restoring homeostasis in fibrotic lung disease.

## Data Availability

The data supporting the results of the current study are available from the corresponding author upon reasonable request.

## Supplementary Materials

The following supporting information can be downloaded at: https://www.mdpi.com/article/10.3390/ijms27094016/s1.

## Abbreviations

The following abbreviations are used in this manuscript:

AA	Arachidonic acid
2-APB	2-aminoethoxydiphenyl borate
ARC	Arachidonate-regulated Ca^2+^ channels
ATP	Adenosine triphosphate
A.U.	Arbitrary units
0Ca^2+^	Calcium-free solution
[Ca^2+^]_i_	Free intracellular calcium concentration
CPA	Cyclopiazonic acid
cPTIO	2-(4-Carboxyphenyl)-4,4,5,5-tetramethylimidazoline-1-oxyl-3-oxide
CRAC	Ca^2+^-release-activated Ca^2+^ channels
DAF-FM	4-amino-5-methylamino-2′,7′-difluorofluorescein diacetate
DAG	Diacylglycerol
*EC*_50_	Half-maximal effective concentration
eNOS	Endothelial nitric oxide synthase
ER	Endoplasmic reticulum
ETYA	Eicosatetraenoic acid
GPN	Glycyl-L-phenylalanine 2-naphthylamide
GPR40	G protein-coupled receptor 40
H_2_DCF-DA	2′,7′-dichlorodihydrofluorescein diacetate
H_2_O_2_	Hydrogen peroxide
iNOS	Inducible nitric oxide synthase
IP_3_	Inositol 1,4,5-trisphosphate
IP_3_Rs	Inositol 1,4,5-trisphosphate receptors
IPF	Idiopathic pulmonary fibrosis
L-NAME	L-N^G^-nitro-L-arginine methyl ester
NAADP	Nicotinic acid adenine dinucleotide phosphate
NAC	N-acetylcysteine
nNOS	Neuronal nitric oxide synthase
NO	Nitric oxide
NOS	Nitric oxide synthase
NOX	Nicotinamide adenine dinucleotide phosphate oxidases
O_2_•^−^	Superoxide anions
ONOO^−^	Peroxynitrite
PA	Palmitoleic acid
PLA_2_	Phospholipase A_2_
PLC	Phospholipase C
PLCβ	Phospholipase C beta
PSS	Physiological saline solution
PTX	Pertussis toxin
R^2^	Coefficient of determination
ROS	Reactive oxygen species
RR	Ruthenium red
Ry	Ryanodine
RyRs	Ryanodine receptors
SEM	Standard error of the mean
SNP	Sodium nitroprusside
SOCE	Store-operated Ca^2+^ entry
STIM	Stromal interaction molecule
TPCs	Two-pore channels 1 and 2
TRPV4	Transient receptor potential vanilloid 4
VOCCs	Voltage-operated calcium channels
