# Supplementary material for: The Ca2+–NO–ROS Crosstalk Induced by Arachidonic Acid in Human Lung Fibroblasts: Implications for Pulmonary Fibrosis
Source: Int J Mol Sci. 2026 Apr 30;27(9):4016. doi: 10.3390/ijms27094016 (PMC13163408; doi:10.3390/ijms27094016)
Supplement: Supplementary file 1 [file ijms-27-04016-s001.zip › Figure S4_proofreading.pdf]

FIGURE S4_Ca0_ATP

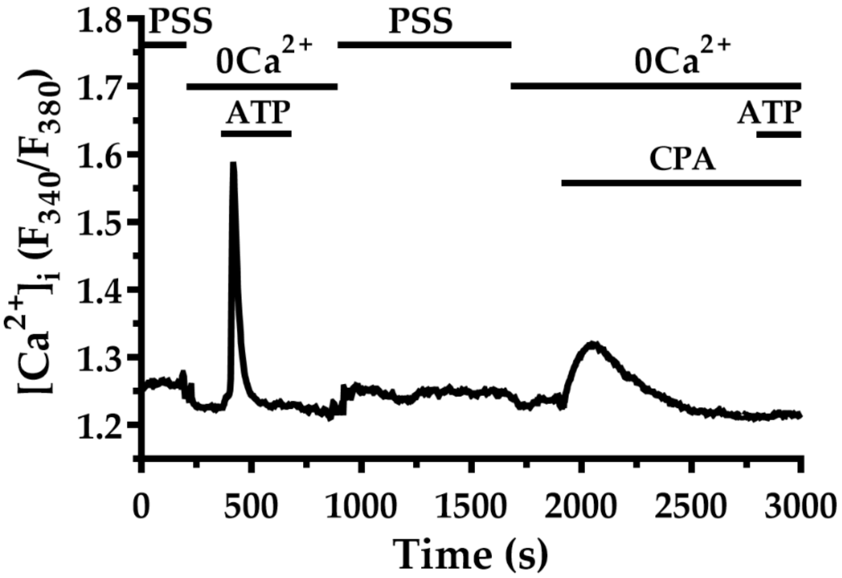

Figure S4. ATP-induced release of ER Ca^{2+} stores in WI-38 human lung fibroblasts under Ca^{2+}-free conditions. Representative trace showing the $[Ca^{2+}]_i$ response to 300 μM ATP in WI-38 cells superfused with a Ca^{2+}-free solution (left). After depletion of the ER Ca^{2+} store using 10 μM cyclopiazonic acid (CPA), ATP failed to elicit a Ca^{2+} response (right). *n*= 51 cells.
