# Supplementary material for: The Ca2+–NO–ROS Crosstalk Induced by Arachidonic Acid in Human Lung Fibroblasts: Implications for Pulmonary Fibrosis
Source: Int J Mol Sci. 2026 Apr 30;27(9):4016. doi: 10.3390/ijms27094016 (PMC13163408; doi:10.3390/ijms27094016)
Supplement: Supplementary file 1 [file ijms-27-04016-s001.zip › Figure S5_proofreading.pdf]

FIGURE S5_VOCCs

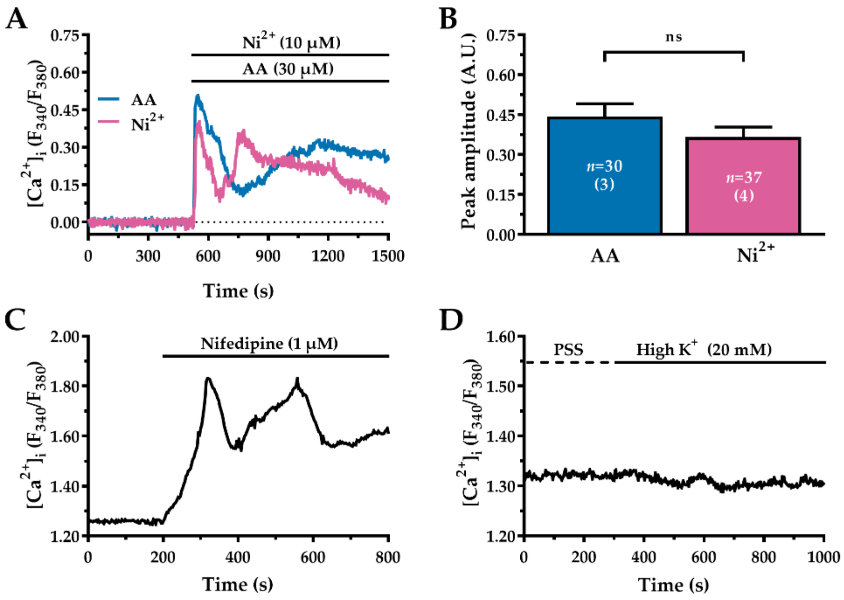

Figure S5. Effect of L-type Ca^{2+} channel blockers on the AA-induced Ca^{2+} response in WI-38 human lung fibroblasts. **A)** Representative traces showing the response to AA (30 μM) in the absence (blue trace) and presence (pink trace) of Ni^{2+} (10 μM). For clarity, the baseline of each Ca^{2+} trace has been normalised to zero. **B)** Mean ± SEM of the peak amplitude of the Ca^{2+} signal induced by AA in the presence or absence of Ni^{2+}. Statistical analysis: Mann–Whitney U test (ns, $p > 0.05$). *n* represents the number of cells analysed. The number of independent experimental replicates is indicated in parentheses. **C)** A representative trace of $[Ca^{2+}]_i$ in WI-38 cells loaded with Fura-2/AM, demonstrating the effect of nifedipine (1 μM). Nifedipine alone elicited a Ca^{2+} response prior to the addition of AA. **D)** Representative trace of $[Ca^{2+}]_i$ in response to high potassium (20 mM) stimulation.
