# Supplementary material for: The Ca2+–NO–ROS Crosstalk Induced by Arachidonic Acid in Human Lung Fibroblasts: Implications for Pulmonary Fibrosis
Source: Int J Mol Sci. 2026 Apr 30;27(9):4016. doi: 10.3390/ijms27094016 (PMC13163408; doi:10.3390/ijms27094016)
Supplement: Supplementary file 1 [file ijms-27-04016-s001.zip › Figure S6_proofreading.pdf]

FIGURE S6_NO acts upstream Ca^{2+} singalling

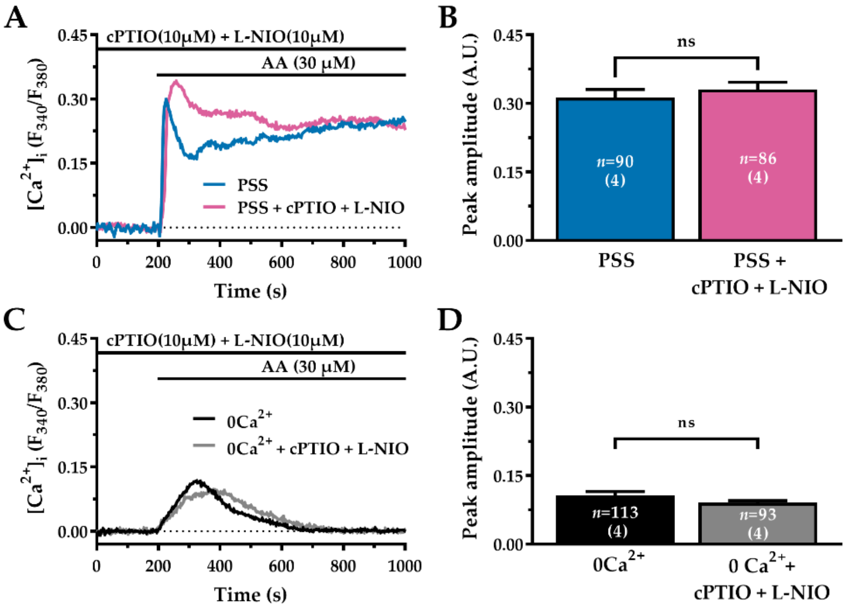

Figure S6. NO does not act upstream of AA-induced Ca^{2+} signals in WI-38 human lung fibroblasts. **A)** Representative traces showing Ca^{2+} responses to AA (30 μM) in PSS (a Ca^{2+}-containing solution) under control conditions (blue trace) and after pre-incubation (60 min) with the eNOS inhibitor L-NIO (10 μM) and the NO scavenger cPTIO (10 μM) (pink trace). The arrow indicates the time of AA addition. For clarity, fluorescence baselines have been normalised to zero. **B)** Quantification of the peak amplitude (Mean ± SEM) for the conditions shown in (**A**), expressed in A.U. **C)** Representative traces showing Ca^{2+} responses to AA (30 μM) in Ca^{2+}-free solution ($0Ca^{2+}$) under control conditions (black trace) and after pre-incubation with L-NIO and cPTIO (grey trace). **D)** Quantification of the peak amplitude (Mean ± SEM) for the conditions shown in (**C**), expressed in A.U. Statistical analysis: Mann–Whitney U test (ns, $p > 0.05$). The *n* value represents the number of cells analysed. The number of independent experimental replicates is indicated in parentheses.
