# Supplementary material for: The Ca2+–NO–ROS Crosstalk Induced by Arachidonic Acid in Human Lung Fibroblasts: Implications for Pulmonary Fibrosis
Source: Int J Mol Sci. 2026 Apr 30;27(9):4016. doi: 10.3390/ijms27094016 (PMC13163408; doi:10.3390/ijms27094016)
Supplement: Supplementary file 1 [file ijms-27-04016-s001.zip › Figure S7_proofreading.pdf]

FIGURE S7_NOX2

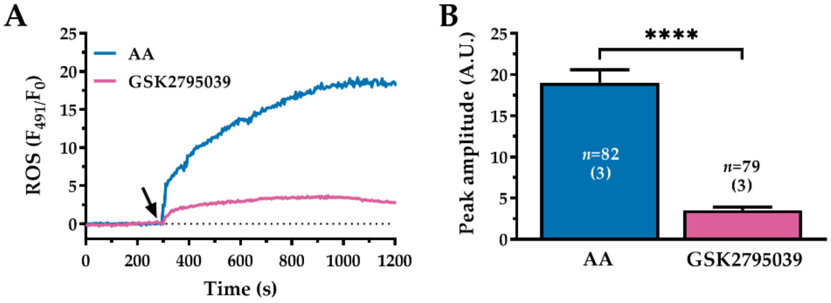

Figure S7. NOX2 mediates AA-induced ROS generation downstream of NO signalling in WI-38 human lung fibroblasts. **A)** Representative traces of ROS production induced by AA (30 μM) in the absence (control, blue trace) and presence of the selective NOX2 inhibitor GSK2795039 (30 μM, 2 h pre-incubation, pink trace). For clarity, fluorescence baselines were normalised to zero. The arrow indicates the time of stimulation **B)** Data are presented as mean ± SEM of the peak response. Statistical analysis: Mann–Whitney U test. ****, $p < 0.0001$. *n* indicates the number of cells analysed. The number of independent experimental replicates is indicated in parentheses.
