# Supplementary material for: The Ca2+–NO–ROS Crosstalk Induced by Arachidonic Acid in Human Lung Fibroblasts: Implications for Pulmonary Fibrosis
Source: Int J Mol Sci. 2026 Apr 30;27(9):4016. doi: 10.3390/ijms27094016 (PMC13163408; doi:10.3390/ijms27094016)
Supplement: Supplementary file 1 [file ijms-27-04016-s001.zip › Figure S8_proofreading.pdf]

FIGURE S8_ NAC experiments

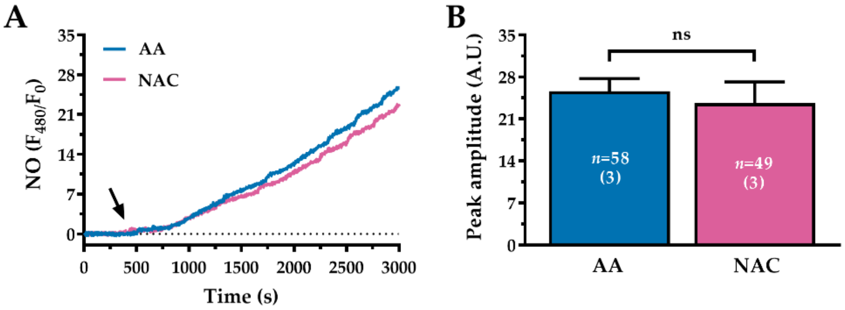

Figure S8. Antioxidant treatment does not affect AA-induced NO production in WI-38 human lung fibroblasts. **A)** Representative traces showing NO production, as measured by DAF fluorescence, in response to AA (30 μM) both in the absence (blue trace) and in the presence (pink trace) of NAC (1 mM, pre-incubated for 60 minutes). The arrow indicates the time of AA addition. For clarity, fluorescence baselines have been normalised to zero. **B)** Quantification of the peak amplitude (Mean ± SEM) for the conditions shown in (**A**), expressed in A.U. Statistical analysis: Mann–Whitney U test (ns, $p > 0.05$). *n* indicates the number of cells analysed. The number of independent experimental replicates is indicated in parentheses.
