# Supplementary material for: The Ca2+–NO–ROS Crosstalk Induced by Arachidonic Acid in Human Lung Fibroblasts: Implications for Pulmonary Fibrosis
Source: Int J Mol Sci. 2026 Apr 30;27(9):4016. doi: 10.3390/ijms27094016 (PMC13163408; doi:10.3390/ijms27094016)
Supplement: Supplementary file 1 [file ijms-27-04016-s001.zip › Figure S9_proofreading.pdf]

# FIGURE S9_GRAPHICAL ABSTRACT

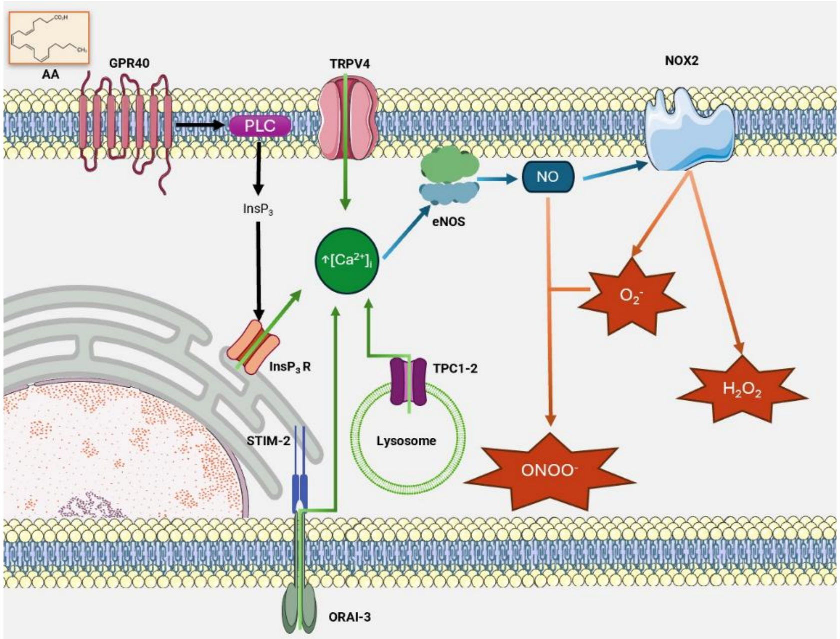


**Figure S9.** Proposed mechanism underlying AA-induced $Ca^{2+}$–NO–ROS signalling in human lung fibroblasts. Binding of AA to GPR40 activates PLC, leading to the generation of the second $IP_3$. $IP_3$ binds to $IP_3Rs$ on the endoplasmic reticulum membrane, triggering an initial wave of $Ca^{2+}$ release from intracellular stores. Depletion of ER $Ca^{2+}$ subsequently activates SOCE through STIM–ORAI signalling, thereby sustaining $Ca^{2+}$ influx from the extracellular space. In parallel, TRPV4 channels provide an additional store-independent pathway for $Ca^{2+}$ entry. The $Ca^{2+}$ signal is further amplified by mobilisation of lysosomal $Ca^{2+}$ stores through TPC1–2. The convergence of $Ca^{2+}$ signals derived from the ER, lysosomes, and the extracellular space results in a robust and sustained elevation of $[Ca^{2+}]_i$. This prolonged $Ca^{2+}$ elevation activates endothelial eNOS, leading to increased NO production. Our pharmacological data indicate that NO generation is required for the subsequent activation of NOX2, resulting in the production of ROS. The main ROS generated downstream of NOX2 include superoxide anion ($O_2\bullet^-$) and hydrogen peroxide ($H_2O_2$). In addition, superoxide may react with NO to form peroxynitrite ($ONOO^-$), further contributing to oxidative and nitrosative stress. Consistent with this mechanism, the fluorescent probe $H_2$-DCF detects hydrogen peroxide and peroxynitrite, but not superoxide, suggesting that the ROS produced in response to AA likely include $H_2O_2$, $O_2\bullet^-$, and $ONOO^-$. Sustained $Ca^{2+}$ elevation and redox imbalance may promote fibroblast activation and contribute to the pathological tissue remodelling associated with pulmonary fibrosis.
