# Supplementary material for: The Ca2+–NO–ROS Crosstalk Induced by Arachidonic Acid in Human Lung Fibroblasts: Implications for Pulmonary Fibrosis
Source: Int J Mol Sci. 2026 Apr 30;27(9):4016. doi: 10.3390/ijms27094016 (PMC13163408; doi:10.3390/ijms27094016)
Supplement: Supplementary file 1 [file ijms-27-04016-s001.zip › Figure S1_proofreading.pdf]

FIGURE S1_WESTERN BLOT

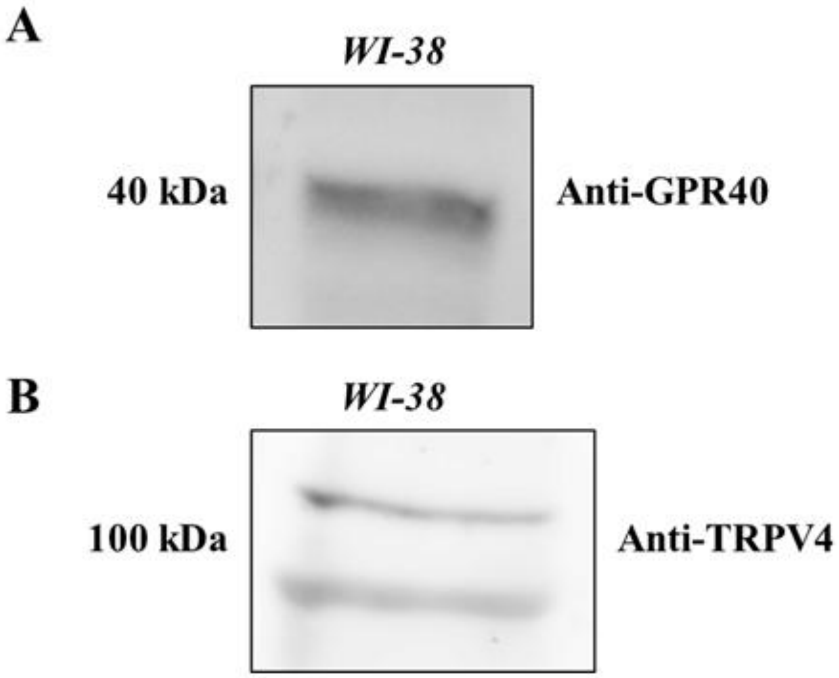

Figure S1. Expression of GPR40 and TRPV4 in WI-38 cells. Representative Western blot analyses performed on WI-38 cell lysates. **A)** A single immunoreactive band at approximately 40 kDa, consistent with the expected molecular weight of the GPR40 receptor. **B)** Detection of a doublet at approximately 100 kDa, corresponding to TRPV4 protein expression.
