# Supplementary material for: The Ca2+–NO–ROS Crosstalk Induced by Arachidonic Acid in Human Lung Fibroblasts: Implications for Pulmonary Fibrosis
Source: Int J Mol Sci. 2026 Apr 30;27(9):4016. doi: 10.3390/ijms27094016 (PMC13163408; doi:10.3390/ijms27094016)
Supplement: Supplementary file 1 [file ijms-27-04016-s001.zip › Figure S2_proofreading.pdf]

FIGURE S2_PLC inhibition in $0Ca^{2+}$

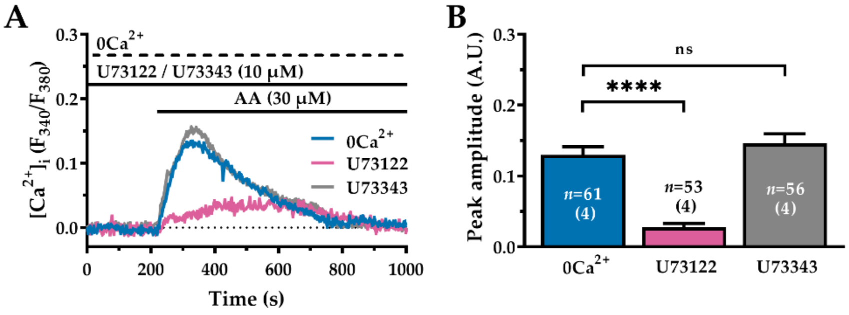

Figure S2. PLC inhibition reduces the AA-induced Ca^{2+} response in a Ca^{2+}-free extracellular medium. **A)** Representative traces of intracellular Ca^{2+} signals recorded in cells stimulated with AA (30 μM) under Ca^{2+}-free conditions ($0Ca^{2+}$, blue trace). The cells were pre-incubated for 30 min with either U73122 (10 μM; pink trace) or its inactive analogue, U73343 (10 μM; grey trace). For clarity, the baseline of the traces has been adjusted to zero. **B)** Summary data (mean ± SEM) for the conditions shown in (**A**), expressed in arbitrary units (A.U.). Statistical analysis was performed using the Kruskal–Wallis test (****, $p < 0.0001$; ns, $p > 0.05$). *n* indicates the number of cells analysed. The number of independent experimental replicates is indicated in parentheses.
