# Supplementary material for: The Ca2+–NO–ROS Crosstalk Induced by Arachidonic Acid in Human Lung Fibroblasts: Implications for Pulmonary Fibrosis
Source: Int J Mol Sci. 2026 Apr 30;27(9):4016. doi: 10.3390/ijms27094016 (PMC13163408; doi:10.3390/ijms27094016)
Supplement: Supplementary file 1 [file ijms-27-04016-s001.zip › Figure S3_proofreading.pdf]

FIGURE S3_Caffeine

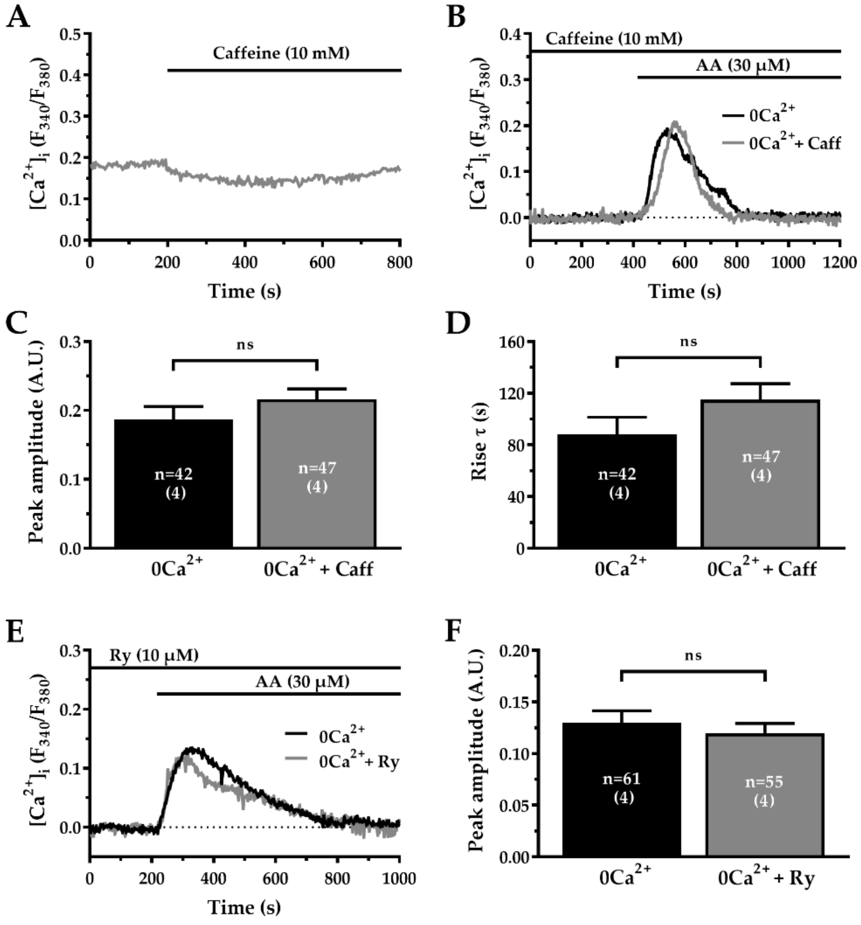

Figure S3. RyRs do not contribute to AA-evoked Ca^{2+} release. **A)** Representative trace showing the response to caffeine (10 mM) in Fura-2/AM-loaded WI-38 cells bathed in PSS. **B)** Representative Ca^{2+} traces evoked by AA (30 µM) under control $0Ca^{2+}$ conditions (black) and after pretreatment with caffeine (10 mM; 10 min, grey). For clarity, the baseline of each Ca^{2+} trace has been normalised to zero. **C**) Quantification of the peak amplitude (mean ± SEM) for the conditions shown in (**B**). **D)** Quantification of the rate of rise of the Ca^{2+} transient t (s) (mean ± SEM) for the conditions shown in (**B**). **E**) Representative Ca^{2+} traces evoked by AA (30 µM) under control $0Ca^{2+}$ conditions (black) and after pretreatment with ryanodine (10 µM; 60 min, grey). For clarity, the baseline of each Ca^{2+} trace was normalised to zero. **F**) Quantification of the peak amplitude (mean ± SEM) for the conditions shown in (**E**). Statistical analysis was performed using the Mann–Whitney U test. ns, $p > 0.05$. *n* indicates the number of cells analysed. The number of independent experimental replicates is indicated in parentheses.
